# The Common Hallmarks and Interconnected Pathways of Aging, Circadian Rhythms, and Cancer: Implications for Therapeutic Strategies

**DOI:** 10.34133/research.0612

**Published:** 2025-03-05

**Authors:** Jie Wang, Fanglin Shao, Qing Xin Yu, Luxia Ye, Dilinaer Wusiman, Ruicheng Wu, Zhouting Tuo, Zhipeng Wang, Dengxiong Li, William C. Cho, Wuran Wei, Dechao Feng

**Affiliations:** ^1^ Department of Urology, Institute of Urology, West China Hospital, Sichuan University, Chengdu 610041, China.; ^2^ Department of Rehabilitation, The Affiliated Hospital of Southwest Medical University, Luzhou 646000, China.; ^3^ Department of Pathology, Ningbo Clinical Pathology Diagnosis Center, Ningbo, Zhejiang 315211, China.; ^4^ Department of Pathology, Ningbo Medical Centre Lihuili Hospital, Ningbo, Zhejiang 315040, China.; ^5^ Department of Public Research Platform, Taizhou Hospital of Zhejiang Province Affiliated to Wenzhou Medical University, Linhai, China.; ^6^ Department of Comparative Pathobiology, College of Veterinary Medicine, Purdue University, West Lafayette, IN 47907, USA.; ^7^ Purdue Institute for Cancer Research, Purdue University, West Lafayette, IN 47906, USA.; ^8^ Department of Urological Surgery, Daping Hospital, Army Medical Center of PLA, Army Medical University, Chongqing, China.; ^9^ Department of Urology, Sichuan Provincial People’s Hospital, University of Electronic Science and Technology of China, Chengdu, China.; ^10^ Department of Clinical Oncology, Queen Elizabeth Hospital, Hong Kong SAR, China.; ^11^ Division of Surgery and Interventional Science, University College London, London W1W 7TS, UK.

## Abstract

The intricate relationship between cancer, circadian rhythms, and aging is increasingly recognized as a critical factor in understanding the mechanisms underlying tumorigenesis and cancer progression. Aging is a well-established primary risk factor for cancer, while disruptions in circadian rhythms are intricately associated with the tumorigenesis and progression of various tumors. Moreover, aging itself disrupts circadian rhythms, leading to physiological changes that may accelerate cancer development. Despite these connections, the specific interplay between these processes and their collective impact on cancer remains inadequately explored in the literature. In this review, we systematically explore the physiological mechanisms of circadian rhythms and their influence on cancer development. We discuss how core circadian genes impact tumor risk and prognosis, highlighting the shared hallmarks of cancer and aging such as genomic instability, cellular senescence, and chronic inflammation. Furthermore, we examine the interplay between circadian rhythms and aging, focusing on how this crosstalk contributes to tumorigenesis, tumor proliferation, and apoptosis, as well as the impact on cellular metabolism and genomic stability. By elucidating the common pathways linking aging, circadian rhythms, and cancer, this review provides new insights into the pathophysiology of cancer and identifies potential therapeutic strategies. We propose that targeting the circadian regulation of cancer hallmarks could pave the way for novel treatments, including chronotherapy and antiaging interventions, which may offer important benefits in the clinical management of cancer.

## Introduction

Cancer, circadian rhythms, and aging are 3 biological processes closely associated with health and disease. While they may appear to be independent, increasing evidence suggests that there are complex interactions among them. The relationship between aging and cancer is very clear. Aging remains to represent the foremost risk factor across various cancer types, correlating with an elevated incidence of cancer that typically reaches its peak around the age of 85 years [1–3]. Reportedly, excluding other objective factors and causes of competitive death, the cumulative risk of developing cancer by the age of 75 reaches as high as 21.4% [4]. Furthermore, for prostate cancer, the incidence of latent prostate cancer, which is defined as prostate cancer detected by autopsy, can be as high as 35.1% to 51% [5,6]. These truths seem that as long as one lives long enough, developing cancer becomes an inevitable occurrence. On the mechanism, aging and cancer share many common hallmarks, including genomic instability, epigenetic alterations, chronic inflammation, cellular senescence, and so on, which serve as intermediaries between aging and cancer [2,7,8].

Similarly, circadian rhythms are 24-h cycles that govern a range of physiological processes in living organisms, such as sleep–wake cycles, hormone release, metabolism, and cell proliferation [9,10]. Disruption of circadian rhythms has also been shown to contribute importantly to the development and progression of cancers, although the exact mechanisms are not yet fully understood [11,12]. Chronic sleep deprivation, insomnia, and shift work have been linked to an increased risk of cancer, especially breast cancer [13,14], prostate cancer [14,15], and colorectal cancer [16,17]. This is connected to the regulation of sleep, immune function, and metabolism by circadian rhythms [12,18]. In 2007, the International Agency for Research on Cancer, part of the World Health Organization, classified circadian rhythm disruption as a probable carcinogen for humans (Group 2A) due to the increased cancer susceptibility observed in individuals engaged in shift work [19]. Mechanistically, circadian rhythm proteins exhibit physical interactions with molecules implicated in cancer-related pathways, thus exerting influence over cancer initiation and progression [20,21]. Concurrently, components of circadian rhythms have the ability to directly or indirectly regulate the expression of numerous genes in different cell types. This regulatory effect extends to key cellular processes, including but not limited to nutrient metabolism, redox balance, autophagy, and DNA damage repair [21,22]. Therefore, circadian rhythm dysfunction is intricately linked to cancer hallmarks.

**Fig. 1.**
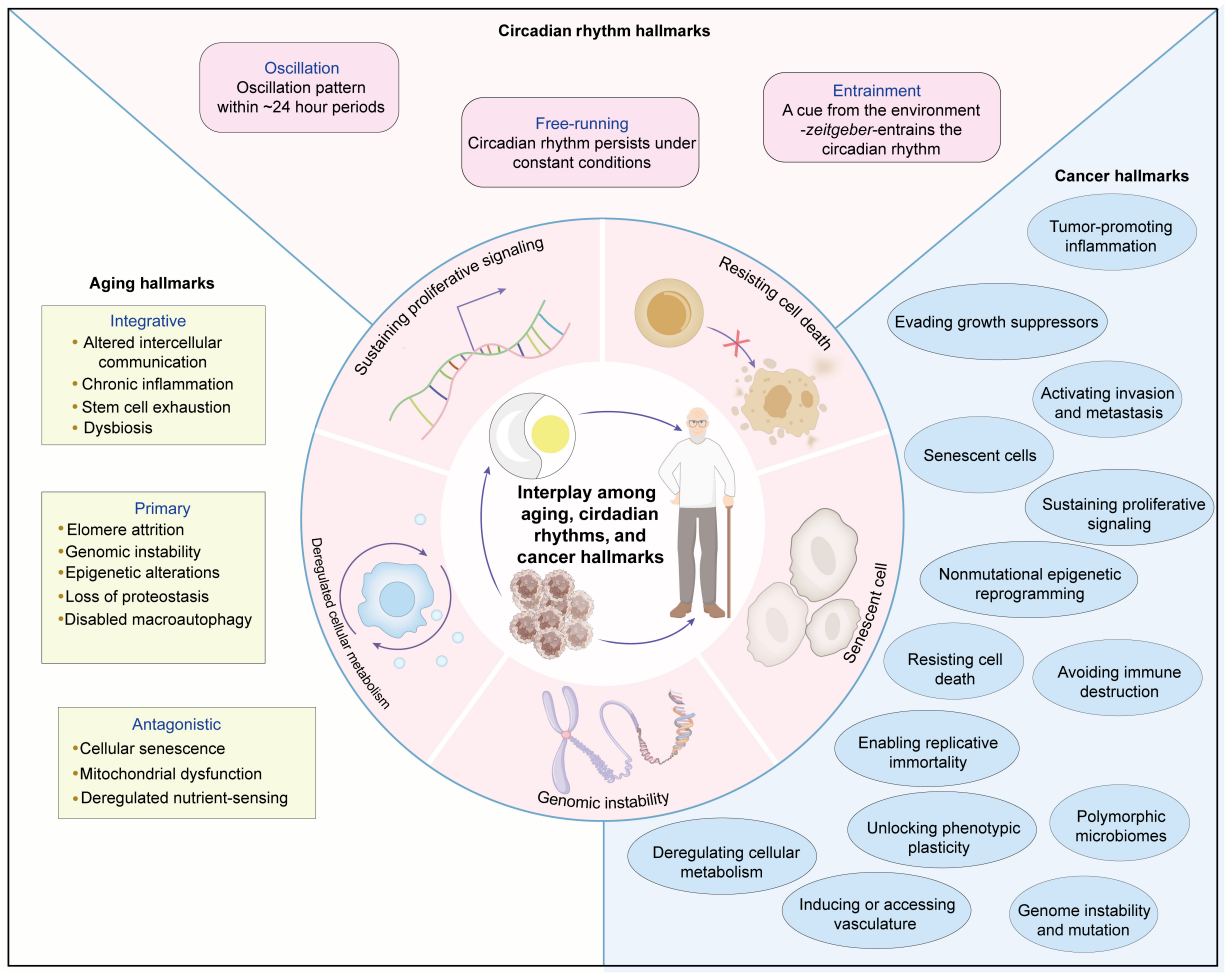
The crosstalk among cancer, circadian rhythms, and aging. This figure illustrates the interplay among circadian rhythms, aging, and cancer hallmarks, highlighting their respective features and interactions. The central illustration represents the interaction between aging, circadian rhythms, and cancer hallmarks. The key hallmarks include sustaining proliferative signaling, resisting cell death, senescent cell, genomic instability, and deregulated cellular metabolism. The left side shows aging hallmarks, including the following: Primary: telomere attrition, genomic instability, epigenetic alterations, loss of proteostasis, and disabled macroautophagy; Antagonistic: cellular senescence, mitochondrial dysfunction, and deregulated nutrient sensing; and Integrative: altered intercellular communication, chronic inflammation, stem cell exhaustion, and dysbiosis. The right side shows cancer hallmarks: tumor-promoting inflammation, evading growth suppressors, activating invasion and metastasis, senescent cells, sustaining proliferative signaling, nonmutational epigenetic reprogramming, resisting cell death, avoiding immune destruction, enabling replicative immortality, unlocking phenotypic plasticity, inducing or accessing vasculature, polymorphic microbiomes, and genome instability and mutation. The top shows circadian rhythm hallmarks: oscillation refers to the ~24-h period oscillation pattern of circadian rhythms, free-running describes how circadian rhythms persist under constant conditions, and entrainment involves cues from the environment ("*zeitgeber*") that synchronize the circadian rhythm.

Furthermore, there also exist complex and multifaceted relationships between aging and circadian rhythms. On the one hand, the aging process reduces the resilience of circadian rhythms, resulting in disrupted sleep–wake cycles, a diminished ability to synchronize circadian rhythms in peripheral tissues, and changes in the molecular functioning of circadian clock outputs [23,24]. On the other hand, circadian rhythm dysfunction can accelerate the aging process by compromising essential bodily functions. These disruptions lead to increased oxidative stress, which refers to cellular damage caused by an imbalance between the production of reactive oxygen species (ROS) and the cell’s ability to neutralize them [25,26]. This imbalance of ROS can lead to DNA damage, protein denaturation, and lipid peroxidation, ultimately contributing to inflammation and the development of age-related health issues [24,27,28]. Numerous hallmarks of aging and cancer, including genomic instability, cellular senescence, deregulating cellular metabolism, and so on, either directly impact the function of the circadian rhythms or are under the regulation of circadian rhythms. Therefore, we review the current understanding of the shared hallmarks and mechanisms between circadian rhythms, aging, and cancer, as well as how the interplay between circadian rhythms and aging influences tumorigenesis and the progression of tumors. Figure 1 shows the interplay among cancer, circadian rhythms, and aging.

## Physiological Mechanisms of Circadian Rhythms

A functional circadian clock is characterized by 3 key features: First, gene expression or protein production must follow a rhythmic oscillation pattern that repeats approximately every 24 h, reflecting the biological processes’ alignment with the day–night cycle. Second, this circadian rhythm is entrained, or synchronized, by an external environmental stimulus known as a *zeitgeber*, with light being the most common, but also including factors like temperature and feeding cycles. Lastly, even after the external stimulus (*zeitgeber*) is removed, the circadian clock continues to maintain its oscillatory rhythm, demonstrating its intrinsic ability to function autonomously over time [29]. At the molecular level, circadian rhythms regulate various biochemical, physiological, and behavioral processes, creating a roughly 24-h cycle through the transcription–translation feedback loop (TTFL) [30] involving core clock genes such as brain and muscle arnt-like (*BMAL1)*, circadian locomotor output cycles kaput (*CLOCK*), period (*PERs*), cryptochrome (*CRYs*), nuclear receptor subfamily 1 group D member (*NR1D1/2*), and retinoic acid-related receptor alpha beta gamma (*RORαβϒ*) [31]. These transcription factors interact with each other in a feedback loop to maintain the circadian rhythms.

**Fig. 2.**
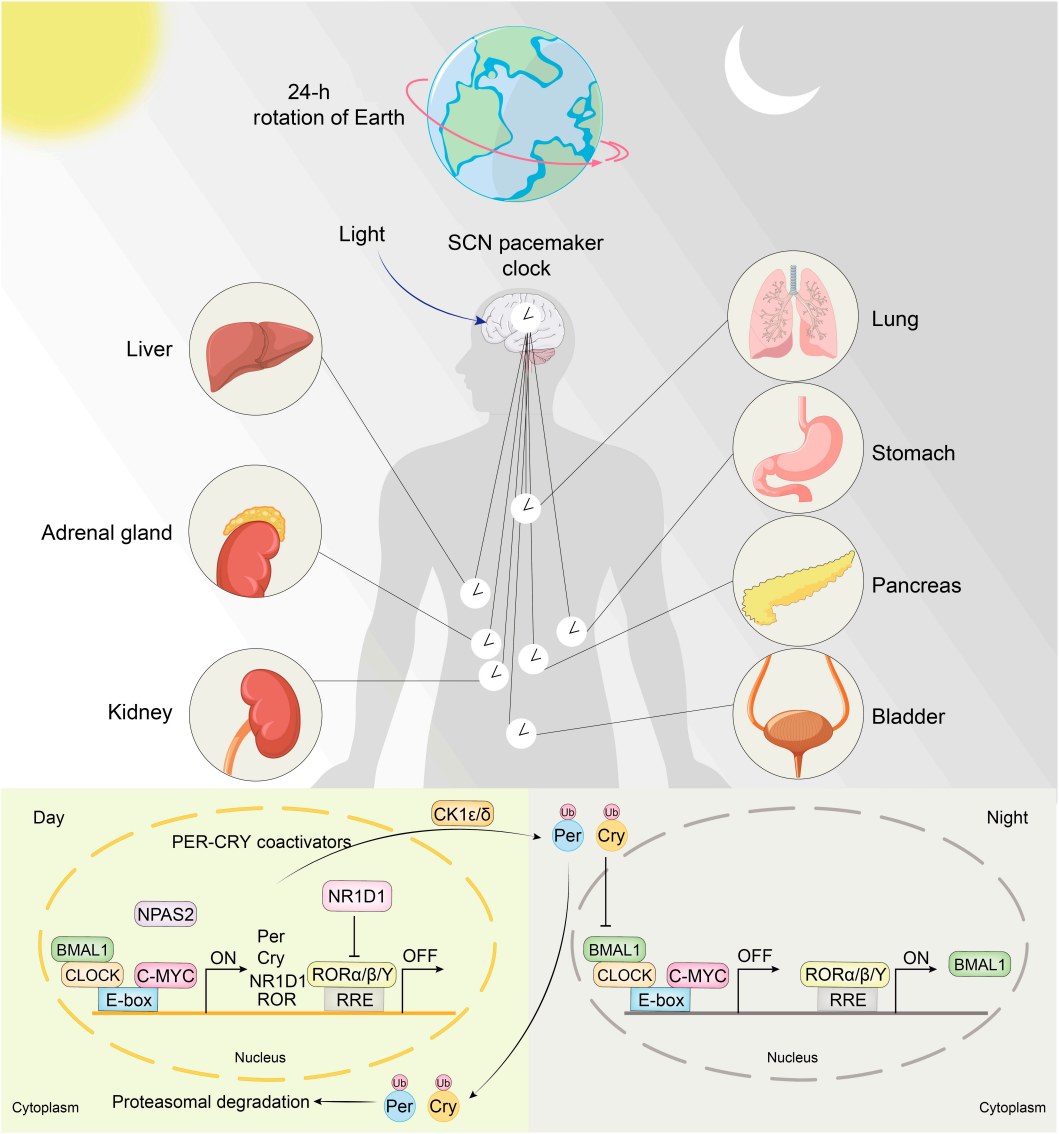
The physiological mechanisms of normal circadian rhythms. The molecular mechanisms of the circadian rhythms, governed by the SCN pacemaker clock, which synchronizes peripheral clocks in various organs such as the liver, adrenal gland, kidney, lung, stomach, pancreas, bladder, and prostate. At the molecular level, the circadian rhythm is driven by transcriptional–translational feedback loops. During the day, the heterodimerization of BMAL1 and CLOCK (or NPAS2) leads to the activation of E-box elements in the promoters of clock-controlled genes, including Per gene and Cry gene. These genes are transcribed and translated into PER and CRY proteins, which accumulate in the cytoplasm. As night approaches, PER and CRY translocate back into the nucleus, where they inhibit the activity of the BMAL1–CLOCK complex, thereby repressing their own transcription and completing the feedback loop. Additionally, other regulatory proteins, such as NR1D1 and RORα/β/γ, modulate the expression of BMAL1, ensuring the rhythmic oscillation of this key clock gene. Posttranslational modifications, including phosphorylation by CK1ε/δ, target PER and CRY for ubiquitination and subsequent proteasomal degradation, thus resetting the clock for the next cycle. This molecular clock machinery coordinates the expression of thousands of genes, impacting diverse physiological processes such as metabolism, hormone release, and cell cycle regulation. The interplay between the central SCN clock and peripheral clocks ensures that the organism’s internal timing aligns with external day–night cycles, promoting optimal function and health. SCN, suprachiasmatic nucleus; BMAL1, brain and muscle arnt-like 1; CLOCK, circadian locomotor output cycles kaput; NPAS2, neuronal PAS domain-containing protein 2; PER, period; CRY, cryptochrome; NR1D, nuclear receptor subfamily 1 group D member; ROR, recombinant receptor tyrosine kinase like orphan receptor; RRE, NR1D/ROR response elements; CK, casein kinase.

CLOCK and BMAL1 form heterodimers and bind to enhancer or “E-box” sequences (CACGTG) in the promoters of PER and CRY genes, stimulating transcription. Subsequently, PER and CRY proteins function as a negative component of the clock by forming heterodimers in the nucleus, thereby repressing CLOCK–BMAL1 activity [32]. The turnover of inhibitory PER and CRY proteins initiates a new cycle driven by CLOCK and BMAL1 through E-box elements. NR1D1/2 and RORα/β/γ play roles in various physiological processes [33]. Another feedback loop involves nuclear orphan receptors RORα, RORβ, and RORγ as activators, and NR1D1 and NR1D2 as inhibitors, controlling the circadian transcription of BMAL1 [34,35]. Furthermore, NR1D and ROR establish feedback loops that regulate BMAL1 expression [36]. The CLOCK/BMAL1 heterodimer enhances NR1D transcription, while a ROR/NR1D-response element-dependent mechanism negatively regulates BMAL1 expression [37]. Figure 2 demonstrates the physiological mechanisms of normal circadian rhythms.

## Circadian Rhythms and Cancer

**Fig. 3.**
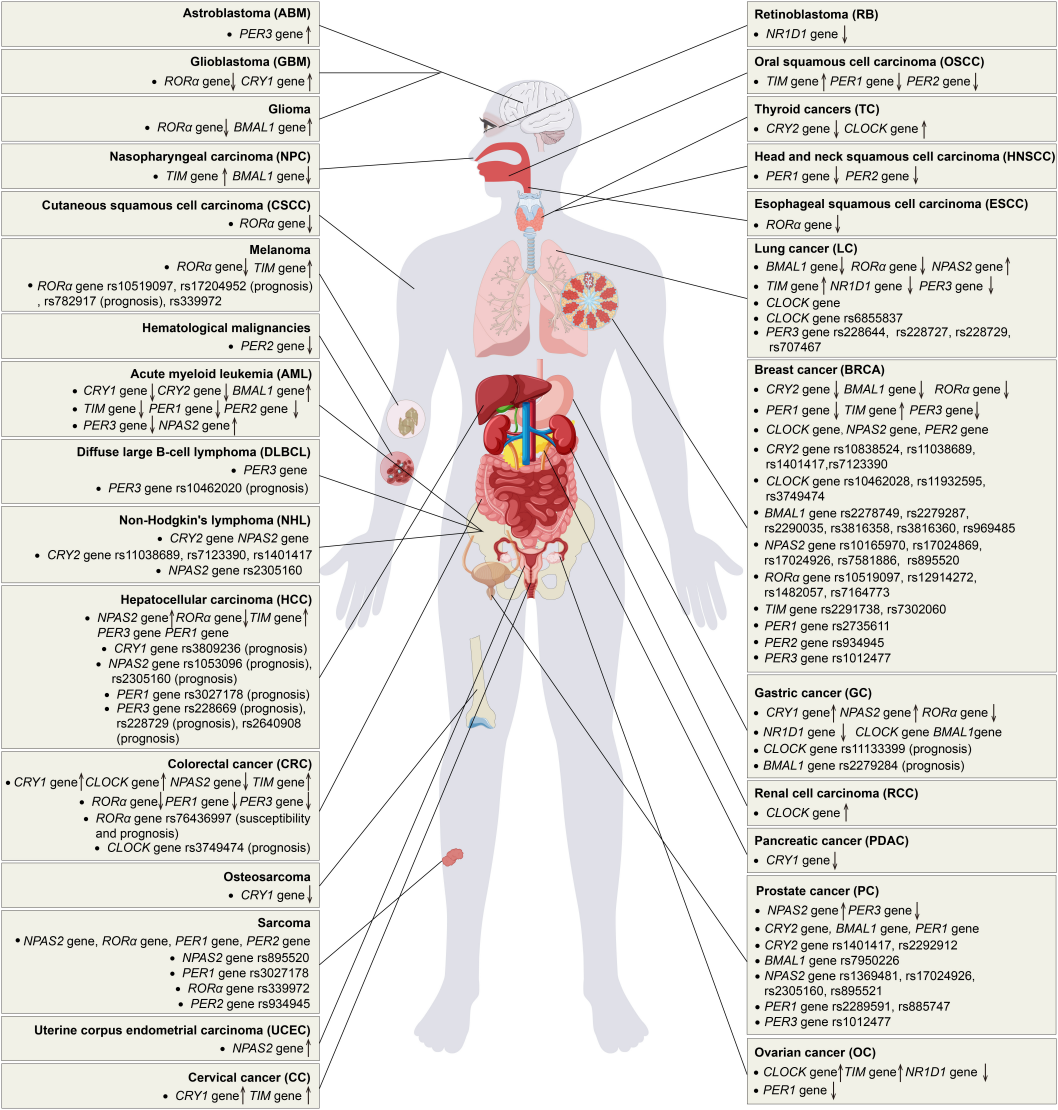
The expression of core circadian genes in different tumors and the SNPs associated with tumor risk and prognosis. This schematic diagram highlights the role of core circadian clock genes (*CRY1*, *CRY2*, *CLOCK*, *BMAL1*, *NPAS2*, *RORα*, *TIM*, *NR1D1*, *PER1*, *PER2*, and *PER3*) in the pathogenesis and prognosis of different cancer types. The expression or dysregulation of these genes is implicated in a variety of malignancies, including but not limited to breast cancer, colorectal cancer, hepatocellular carcinoma, glioblastoma, and hematologic malignancies. Genes involved in circadian regulation are indicated as either up-regulated (↑) or down-regulated (↓) depending on their relationship with specific cancer types. Polymorphisms associated with prognosis or susceptibility to cancer are also noted. SNPs, single-nucleotide polymorphisms.

Studies have demonstrated that disturbances in circadian rhythm genes can increase the likelihood of proliferation, invasion, and migration in various types of cancer such as breast cancer [38], colon cancer [39], hepatocellular carcinoma [40], melanoma [41], and ovarian cancer [42]. Researches have increasingly concentrated on comparing the expression levels of circadian core genes in tumor and normal tissues to better understand the potential connection between circadian rhythm disturbances and cancer. In this review, we systematically summarized the expression of 11 circadian core genes across various types of tumors, including *CRY1* [43–49], *CRY2* [49–51], *CLOCK* [51–54], *BMAL1* [55–59], *NPAS2* [60–66], *RORα* [67–76], *TIM* [49,69,77–84], *NR1D1* [85–88], *PER1* [49,89–93], *PER2* [49,94–96], and *PER3* [49,97–101]*.* In addition, many studies highlight that single-nucleotide polymorphisms (SNPs) of core circadian genes are associated with an increased risk of various tumors and their prognosis. In this review, we systematically summarized the relationship between different SNPs in 10 core circadian genes and the risk and prognosis of various tumors, including *CRY1* (rs3809236 [102]), *CRY2* (rs10838524 [103], rs11038689 [50,104], rs1401417 [50,104,105], rs2292912 [106], rs7123390 [50,104]), *CLOCK* (rs10462028 [107], rs11133399 [108], rs11932595 [109], rs3749474 [110,111], rs6855837 [112]), *BMAL1* (rs2278749 [111], rs2279284 [108], rs2279287 [103], rs2290035 [111], rs3816358 [107], rs3816360 [113], rs7950226 [106], rs969485 [111]), *NPAS2* (rs10165970 [114], rs1053096 [115], rs1369481 [106], rs17024869 [114], rs17024926 [106,111], rs2305160 [105,115,116], rs7581886 [114], rs895520 [114,117], rs895521 [106]), *RORα* (rs10519097 [114,118], rs12914272 [109], rs1482057 [109], rs17204952 [119], rs339972 [117,118], rs7164773 [114], rs76436997 [120], rs782917 [119]), *TIM* (rs2291738 [121], rs7302060 [121]), *PER1* (rs2289591 [106], rs2735611 [103], rs3027178 [102,117], rs885747 [106]), *PER2* (rs934945 [103,117]), and *PER3* (rs1012477 [106,111], rs10462020 [122], rs228644 [112], rs228669 [102], rs228727 [112], rs228729 [102,112], rs2640908 [102], rs707467 [112]). Figure 3 illustrates the expression of core circadian genes in different tumors and the SNPs associated with tumor risk and prognosis.

Circadian rhythm genes can influence various aspects of cancer biology, including proliferation, DNA repair, apoptosis, and stem cell regulation [123–126]. The cell cycle is regulated by cyclin/cyclin-dependent kinase (CDK) complexes, with many genes controlling key steps in cell cycle phases being influenced by the circadian rhythms [123]. For example, c-MYC and cyclin D1, involved in the DNA synthesis phase (S phase), exhibit circadian expression cycles through CLOCK/BMAL1-dependent transactivation [127,128]. Knockdown of BMAL1/CLOCK led to the down-regulation of Wee1, which activated apoptosis and up-regulated p21, resulting in cell cycle arrest at the G2/M phase. The combined effects of Wee1 and p21 contribute to tumor cell death [123]. In addition to these factors, circadian rhythms also control several other factors that regulate the cell cycle, including checkpoint regulators like p16INK4a, p27, p57, and CDK1/cyclin B1, as well as components of cell cycle signaling pathways such as MAPK, Wnt/beta-catenin, and TGF, thereby coordinating the daily rhythm of cell proliferation [129].

The accumulation of DNA damage and the failure of DNA repair mechanisms can lead to apoptosis. The *TP53* gene, which encodes the cellular tumor antigen p53, is the most mutated gene in human cancers [130]. p53 plays a key role in the cellular response to DNA damage by regulating the DNA damage checkpoint, controlling cell cycle arrest, and inducing apoptosis [129]. BMAL1 and PER2 tightly regulate the transcription, stability, and activity of p53 [131,132]. BMAL1 down-regulation hinders p53-dependent p21 induction, while the absence of PER2 impairs p53 activation in response to DNA damage [133]. Mouse double minute 2 homolog (MDM2) is a crucial negative regulator of p53, maintaining its cellular levels low in normal cells. PER2 interacts with MDM2, shielding p53 from MDM2-mediated degradation [134,135]. Changes in PER2 levels can impact p53 levels, and PER2 overexpression can affect both p53 protein stability and the transcription of p53 target genes [135,136]. Additionally, p53 inhibits the binding of BMAL1/CLOCK to the PER2 promoter, resulting in the suppression of PER2 expression [137]. Hence, circadian rhythm genes play an important role in regulating p53 at multiple levels.

DNA repair mechanisms encompass direct repair, base excision repair, nucleotide excision repair, double-strand break repair, and cross-link repair. Recent studies suggest that circadian rhythms regulate all of these repair processes [138]. Nucleotide excision repair is a critical mechanism that fixes a broad range of base lesions. In humans, this process involves 6 key factors: RPA, XPA, XPC-HR23, GTF2H1, ERCC5, and XPF-ERCC1. The circadian rhythms directly influence nucleotide excision repair through the repair factor XPA [139], which is activated by CLOCK/BMAL1 and inhibited by CRY/PER [140,141]. Additionally, the circadian rhythms directly regulate double-strand breaks. Through the BMAL1–CLOCK–H4Ac axis, it facilitates DNA end resection to generate single-stranded DNA, thereby promoting homologous recombination. The depletion of BMAL1 significantly enhances the sensitivity of adrenocortical carcinoma to DNA damage-based therapies [124].

Cancer stem cells (CSCs) are a type of cancer cell capable of self-renewal and differentiation into various cell types within a tumor [142,143]. They are crucial in tumor recurrence, metastasis, chemoresistance, and mortality. In cancers like glioblastoma [144], prostate cancer [145], and breast cancer [146], disturbances in circadian rhythm genes can affect the immune response, metabolism, and survival of CSCs [147]. For example, reducing the expression of BMAL1 or CLOCK in glioblastoma stem cells (GSCs) can induce cell cycle arrest and trigger apoptosis [147]. Additionally, CLOCK and BMAL1 in GSCs play a role in immunosuppression in glioblastoma [144]. Conversely, overexpression of PER2 can induce stasis in GSCs, inhibiting their proliferation, migration, and invasiveness [148].

## Circadian Rhythms and Aging

Although the total number of neurons in the suprachiasmatic nucleus (SCN) remains unchanged with age, the strength of the SCN firing rhythm decreases [149]. As the human body ages, the synchronization between the central biological clock and peripheral clocks also changes [30]. The expression and rhythms of core circadian genes, along with their downstream regulatory genes, are disrupted with age, potentially affecting aging at the cellular, tissue, and organismal levels. This includes processes such as immune cell senescence and immunosenescence [24,150]. Conversely, aging may also affect the rhythms at these levels. There is an inseparable interaction between the two, collectively leading to the decline of bodily functions and the occurrence of diseases, including tumors [24,151].

The chronotype changes as individuals age, with the timing of sleep onset and awakening shifting earlier, resulting in a morning chronotype [152]. Older adults generally have an earlier circadian rhythm compared to younger adults, typically by 1 to 2 h. Aging individuals may experience disruptions to their circadian rhythms, leading to more fragmented sleep patterns. Older individuals often show lower levels of daytime activity, poorer sleep quality, and longer periods before falling asleep. Additionally, older adults are more likely to be awakened during the night compared to younger individuals [153]. The circadian clock located in the hypothalamus SCN is responsible for controlling melatonin secretion. This secretion tends to decrease with age, leading to a smaller and earlier peak at night in older adults [154]. Similarly, cortisol rhythms also undergo changes with age, exhibiting a reduction in amplitude and an earlier peak [155].

Aging alters circadian rhythms at the molecular level, with both behavioral and molecular changes driven by the body clock, which, in turn, accelerate the aging process. Melatonin plays a role in various aging processes by protecting the body against oxidative stress, preserving mitochondrial function, and impacting sirtuin 1 levels [156]. Glucocorticoids influence energy and lipid metabolism, inflammation, and cell proliferation. Metabolic rhythms tend to weaken with age, potentially contributing to the increased susceptibility to metabolic disorders in older individuals, such as diabetes, dyslipidemia, and hypertension [157]. Additionally, as immune function decreases with age, there is a gradual activation of a chronic, low-grade proinflammatory process in aging organisms, which may increase the risk of chronic conditions like metabolic syndrome and neurodegenerative diseases. Notably, 2 recent studies indicate that the synergistic interaction between the central biological clock and peripheral clocks can prevent muscle aging and improve muscle function. Kumar et al. [158]. found that in mouse models, restoring circadian rhythms can mitigate the loss of muscle mass and strength, thereby enhancing motor function that declines with age. Mice that had both their central and peripheral clocks restored simultaneously experienced the most significant improvements. This demonstrates the essential communication between the central clock in the brain and the peripheral clocks in muscles, highlighting the importance of their synchronization for maintaining muscle function and preventing premature muscle aging. Mortimer et al. [159]. demonstrated that the peripheral clock in skin tissue can integrate and even override signals from the central clock in the brain by constructing a minimal circadian interaction network in mice with only 2 nodes. This coordination is crucial for maintaining the normal circadian rhythms of epidermal tissue. These results suggest new knowledge and strategies for addressing age-related decline in physical function through circadian rhythm regulation.

## Aging and Cancer

Aging is a complex process that impacts multiple biological systems, resulting in a gradual deterioration of physiological functions [7]. Its impact on cancer development and progression is profound and complex, intertwining at the molecular, cellular, and systemic levels [160,161]. As one of the key risk factors for cancer, it is widely recognized that the incidence of cancer significantly increases with age [2,162,163]. The study indicates that between 1980 and 2000, the population of the United States grew by 23% (from 227 million to 279 million), while the annual incidence of cancer rose by 66% (from 807,000 to 1.34 million) [164]. The increase in cancer incidence was driven primarily by cases diagnosed in patients ≥65 years of age. Moreover, this trend was consistent across different cancer types and populations, indicating an intrinsic link between the aging process and cancer development [164]. This pattern suggests that the cumulative effect of prolonged exposure to carcinogens, along with physiological changes inherent to the aging process, significantly influences cancer risk [164,165].

Several hallmarks of aging, such as genomic instability, epigenetic alterations, cellular senescence, and chronic inflammation, are similar and partially related to the hallmarks of cancer in some aspects [2]. These hallmarks highlight the mechanisms through which aging influences tumor characteristics, emphasizing the intertwined nature of aging and cancer development.

Both aging and cancer are characterized by increased genomic instability and mutation. Genomic instability refers to the increased tendency of genome to acquire mutations, chromosomal rearrangements, or changes in chromosome number over time. This instability leads to alterations and damage to the genetic material, which can result in cellular dysfunction, tumorigenesis, and the progression of various diseases [166,167]. DNA damage accumulates with age, due in part to a decline in repair mechanisms and the natural wear and tear on the genome [168]. Cancer exploits this instability, with mutations in key genes driving uncontrolled cell growth. Cancers often show genomic instability, including significant chromosomal changes and minor nucleotide alterations, which drive malignant cell transformation [166,167]. For example, a recent study revealed that mitochondria-derived ROS disrupt the structural integrity of micronuclei in aggressive cancers by inducing CHMP7 accumulation and oligomerization, which interferes with its interaction with other ESCRT-III components. This disruption leads to the rupture of micronuclear envelopes and promotes chromosomal shattering. The findings also link these ROS-induced changes to tumor hypoxia, thereby connecting the cellular stress from hypoxia to critical mechanisms that drive cancer progression [169]. While organisms have developed intricate DNA repair systems to counteract genetic damage and preserve cellular balance, these processes are not infallible and decline in efficiency with age [170]. Women with germline mutations in BRCA1 or BRCA2 have breast epithelium that is more susceptible to accelerated aging. These mutations also increase their risk of developing breast and ovarian cancer due to impaired DNA repair mechanisms [171]. This gradual decline contributes to increased cancer risk and other age-related diseases due to the steady buildup of genomic damage.

Chronic inflammation is a hallmark of aging, which also contributes to the development and progression of cancer. Inflammation results from various aging-related deficits at molecular, cellular, and systemic levels. Genomic instability, for example, can lead to changes in blood cell populations, increasing pro-inflammatory types that accelerate cardiovascular aging [172]. Additionally, aging-related epigenetic changes, loss of proteostasis, and impaired macroautophagy can lead to the overexpression of pro-inflammatory proteins [7]. Furthermore, aging disrupts nutrient-sensing pathways through overactive growth and insulin signals, exacerbating inflammation [7]. Moreover, as individuals age, senescent cells accumulate and secrete pro-inflammatory and tumor-promoting factors. While cellular senescence acts as a protective mechanism against cancer by halting the proliferation of damaged cells [173], the senescence-associated secretory phenotype (SASP) can create a microenvironment that promotes tumor progression [174]. Studies have shown that inflammatory environment can promote genetic instability, tumor cell proliferation, and metastasis [175]. Chronic inflammation associated with aging may play a key role in the development and progression of cancer.

Aging and cancer also share multiple epigenetic changes. Human DNA undergoes age-related changes, including widespread hypomethylation and hypermethylation of tumor suppressor genes, significantly affecting the epigenetic landscape in aging and cancer [176]. These alterations, particularly in introns or intergenic areas, lead to the silencing of crucial oncosuppressor genes like p16 and p53, fueling tumor development [177]. Targeting epigenomic changes could slow the epigenetic clock and combat hematological cancers. DNMT3A and TET2, pivotal in methylation and demethylation, are often mutated in conditions predisposing individuals to hematologic cancers and heart disease [172]. Chromatin remodeling is also an important part of epigenetic alterations. Factors involved in chromatin remodeling include HP1a, SWI/SNF complex members, and polycomb protein group, which play important roles in antiaging and cancer processes [178]. Lack of function of these proteins results in disordered chromatin structure, manifested by extensive heterochromatin loss and rearrangements. For example, alterations in the SWI/SNF complex are found in up to 25% of human cancer cases [179]. Noncoding RNAs (ncRNAs), including long noncoding RNAs (lncRNAs), microRNAs (miRNAs), and circular RNAs [180,181], play a crucial role in aging and cancer by regulating key elements of the pathways involved in longevity and tumor formation at the posttranscriptional level [182]. Research involving gain- and loss-of-function experiments in both cell cultures and animal models has established the direct impact of ncRNAs, particularly miRNAs, on the processes of aging and cancer development [183]. These alterations affect gene activity and critical cellular functions, playing a marked role in the progression of aging and cancer.

Metabolism is a fundamental aspect of life, and the aging process leads to a decline in bioenergetic function, manifested by disturbances in lipid synthesis and breakdown, as well as glucose utilization [184]. Nicotinamide adenine dinucleotide (NAD^+^) is a crucial coenzyme involved in redox reactions and plays an essential role in energy metabolism [185]. Starting after puberty, NAD^+^ shows an age-related decline [186]. The loss of NAD^+^ caused by aging can damage the function and homeostasis of mitochondria [187]. In addition, the aging process is accompanied by a decrease in mitochondrial transport capacity [188]. Decreased NAD^+^ levels and mitochondrial dysfunction accelerate the aging process. Key NAD^+^ enzymes, including de novo synthesis–nicotinate phosphoribosyl transferase and salvage synthesis–nicotinamide phosphoribosyl transferase, are overexpressed in various cancers. Their overexpression is linked to increased glycolytic activity, cancer progression, chemotherapy resistance, and poor prognosis [188,189]. Mitochondrial damage and dysfunction, including increased mitochondrial fission, decreased mitochondrial fusion, mutations and depletion of mitochondrial DNA, and disruption of mitochondrial proteins, are frequently observed in various cancers [190,191]. These impairments and dysfunctions lead to cancer proliferation, metastasis, invasion, and resistance to treatment [192,193].

Cellular senescence is a key factor in the aging process and contributes to age-related disorders. As individuals age, senescent cells accumulate in aging tissues and in areas affected by age-related diseases, such as osteoarthritis and atherosclerosis [194,195]. These cells can affect the normal functioning of tissues, leading to a gradual decline in their performance. Cellular senescence plays crucial roles at various stages of tumorigenesis, including tumor initiation, progression, and immune escape [196]. The process of senescence triggered by oncogene activation is known as oncogene-induced senescence (OIS). BRAF mutations commonly seen in melanoma initially promote rapid cell growth in melanocytes. This growth is soon limited by OIS, where cells cease to divide and age, often forming benign skin tumors (melanocytic nevi). However, the loss of specific tumor-suppressor genes or proteins, such as PTEN or IGFBP7, can disrupt this senescent state, leading to the progression from benign tumors to malignant melanoma [197,198]. Mutations in the *NF1* gene, associated with type I neurofibromatosis, activate the N-RAS pathway, leading to senescence in both central and peripheral nervous system tumors, marked by high levels of senescence markers like SA-Gal and p16INK4a [199]. Similarly, inactivation of the *VHL* gene, an important tumor suppressor, triggers cellular senescence and benign tumor formation in kidneys through mechanisms involving pRB and p27 [200]. Additionally, deletion of the RB1 gene in thyroid cells activates senescence through the N-RAS pathway, initially leading to benign adenomas that will progress to cancer upon inactivation of the RAS pathway [201]. Moreover, reactivating the tumor suppressor gene p53 in p53-deficient tumors can induce senescence and cause tumor regression in certain cancers like lymphoma and sarcoma [202]. Collectively, these results emphasize that cellular senescence is a critical barrier against tumor progression and that disruption of the balance between cell proliferation and senescence generally leads to the development of malignant tumors.

## Common Hallmarks and Mechanisms among Aging, Circadian Rhythms, and Cancer

### Aging, circadian rhythms, and tumorigenesis

Tumorigenesis is a complex process influenced by multiple factors, including genetic, environmental, and immunological factors. Interactions between these factors can disrupt cellular homeostasis, leading to uncontrolled cell growth and the formation of tumors [8,203]. Understanding the underlying mechanisms of tumorigenesis is crucial for developing effective prevention and treatment strategies tailored to individual patients and tumor types. Several studies have shown a link between shift work and an increased risk of breast cancer in various occupational groups [14,204–207]. However, the mechanisms underlying the association between night shift work and increasing cancer risk remain largely elusive.

Telomere shortening may be one of the reasons why shift work increases the risk of breast cancer. Telomeres are structures located at the ends of chromosomes, consisting of DNA sequences and associated proteins. Their primary function is to protect the chromosomes from damage and instability, as well as to regulate processes such as cell proliferation and aging [208]. With the shortening of telomeres, chromosome instability escalates, leading to cellular senescence and apoptosis [209]. Employment schedules involving night shifts have demonstrated an impact on telomere length [210,211]. Using qPCR in DNA, Samulin Erdem et al. [212]. assessed telomere length of 563 breast cancer patients and 619 controls. They found that working 6 consecutive night shifts for more than 5 years was associated with shorter telomere lengths. In addition, telomere shortening has been linked to increasing breast cancer risk among individuals engaged in prolonged consecutive night shifts. Furthermore, many researchers also utilize various animal models to investigate the mechanisms by which circadian rhythms influence aging and tumorigenesis. Anisimov et al. [213–218]. demonstrated through a series of rat experiments that circadian rhythm disruption could impact the lifespan of rats and significantly accelerate the development of spontaneous tumors and metabolic syndrome. They divided the rats into 3 groups: those exposed to standard, natural light conditions for Northwestern Russia, and constant illumination. They found that compared to the control group, rats under constant or natural light conditions exhibited accelerated aging, with significantly reduced average and maximum lifespans, as well as faster development of spontaneous tumors. The use of the Ala-Glu-Asp-Gly peptide (Epithalon) and melatonin was shown to alleviate the adverse effects of circadian rhythm disruption on the lifespan and tumor development of rats to some extent [215,216,219]. In addition, exposure to constant illumination can disrupt the levels of superoxide dismutase and catalase, 2 essential antioxidant enzymes [220]. Antioxidants play a crucial role in cellular defense by neutralizing and eliminating free radicals, such as superoxide radicals and hydrogen peroxide, which can otherwise cause significant oxidative damage to cells [221,222]. Superoxide dismutase catalyzes the dismutation of superoxide radicals (O2•−) into hydrogen peroxide (H_2_O_2_) and oxygen (O_2_), while catalase further breaks down hydrogen peroxide into water (H_2_O) and oxygen, preventing the accumulation of hydrogen peroxide and its potential cytotoxic effects [221,223]. Through these mechanisms, antioxidant enzymes help maintain the cellular redox balance, reducing oxidative stress and protecting cells from damage and apoptosis [224,225]. However, constant illumination can disrupt this balance by decreasing the activity of these antioxidant enzymes, exposing cells to excessive oxidative stress, which may accelerate aging processes and increase the risk of cancer. The disruption of antioxidant enzymes caused by circadian rhythm disruption may be a potential mechanism through which constant illumination exposure accelerates aging and tumorigenesis in rats. Moreover, at the cellular and molecular level, the different components of circadian rhythms have varying effects on aging and tumors. Katamune et al. [226]. reported distinct roles of negative and positive transcriptional regulators within the circadian feedback loop in oncogene-induced neoplastic transformation. They revealed that deficiency in negative regulators, such as *PER2* and *CRY1/2*, increases susceptibility to transformation by suppressing cell senescence-associated proteins through ATF4 induction, while deficiency in positive regulators like *BMAL1* and *CLOCK* confers resistance to transformation by maintaining the expression of these senescence-associated proteins. In addition, Hashikawa et al. [227] also demonstrated that mice with a mutated CLOCK gene were protected against tumorigenesis induced by chemical carcinogens, by inhibiting the proliferation signals mediated by the epidermal growth factor (EGF) receptor. While wild-type mice developed significant tumors upon exposure to 7,12-dimethylbenzαanthracene (DMBA), chemically induced tumorigenesis was alleviated in *CLOCK* mutated mice. Despite similar levels of DMBA-induced DNA damage in both groups, *CLOCK* mutated mice did not exhibit EGF receptor-mediated RAS activation, which was associated with the expression of the cellular senescence factor p16INK4a. The research of Antoch et al. [228]. also supports this finding. This study reveals that mice with a functional deficiency of CLOCK (*CLOCK*/*CLOCK* mutant mice) do not exhibit an increased predisposition to tumorigenesis, even when challenged by γ-radiation. Instead, they demonstrate high apoptotic rates and low proliferation rates in lymphoid tissues, suggesting a protective effect against cancer development. However, CLOCK mutant mice exhibit an accelerated aging process in response to low-dose irradiation, displaying phenotypes similar to those seen in BMAL1-deficient mice. Research suggests that BMAL1 helps maintain genomic stability by suppressing transposable elements like LINE1 and reducing cellular senescence. A deficiency in BMAL1 can result in genomic instability and heightened oxidative stress, which, in turn, accelerates cellular senescence [229]. Additionally, BMAL1 plays a crucial role in regulating cell proliferation, metabolism, and DNA repair to prevent tumorigenesis [230]. When BMAL1 expression or function is disrupted, it can lead to cell cycle imbalance, DNA damage accumulation, and increased oxidative stress, thereby promoting cancer development [231,232]. The differential roles of BMAL1 in various cancers and cellular senescence, along with the complexity of its regulatory network, make it a potential target for anticancer therapies. In summary, these findings emphasize the complex relationship between circadian disruption, aging, and tumorigenesis, highlighting the interplay of circadian clock components.

### Aging, circadian rhythms, and tumor proliferation and apoptosis

The most fundamental hallmarks of cancer cells is their ability to sustain chronic proliferation [8,203]. In normal tissues, cells carefully control the generation and release of signals that promote growth, which guide the entry and progression of the cell growth and division cycle. However, in tumor tissues, cancer cells obtain the ability to continuously promote their own growth signals, leading to uncontrolled proliferation. In addition, apoptosis or programmed cell death acts as an inherent barrier against cancer progression. Evading apoptosis plays a critical role in tumor development by allowing abnormal cells to proliferate without restraint [203,233]. Aging may affect tumor sustaining proliferative signal through several mechanisms. For example, following the emergence of cellular senescence, it can inhibit tumor cell proliferation by inducing cell cycle arrest and secreting SASP factors. However, it may also promote tumor growth by altering immune regulation, modulating gene expression, and influencing the tumor microenvironment [2,7,234]. This highlights the complex and important regulatory role of cellular senescence in tumor development. The influence of circadian rhythms on sustaining proliferative signaling and evading apoptosis is multifaceted. The cell cycle within organisms is regulated by circadian rhythms. This means that cellular processes such as DNA synthesis and division are influenced by internal circadian rhythms, thereby affecting the rate and frequency of cell proliferation. In many organisms, there is a tendency for DNA replication to be more active during the night [235,236]. This temporal preference ensures that cells divide within a designated time frame, effectively restraining unregulated cell proliferation. The phenomenon of nocturnal DNA replication suggests that the evolution of the circadian rhythms may have been driven by the need to shield DNA from potential damage caused by UV light exposure [237]. Additionally, circadian rhythms regulate the secretion of hormones like growth hormone, insulin, and thyroid hormones, which are crucial for cancer cell proliferation [238–240]. Some hormones may promote cell growth and division, while others may inhibit proliferation. For example, some laboratory and animal studies have shown that melatonin may have a certain inhibitory effect on cancer development. This is because melatonin possesses antioxidant and immune-modulating properties, which can reduce the generation of free radicals, decrease cellular damage, and enhance the immune function, potentially inhibiting the growth and spread of tumor cells [241,242]. Recent studies have also elucidated a direct relationship between the fundamental circadian rhythms and apoptosis. Circadian factors can exhibit dual roles in modulating apoptosis, either promoting or restricting it, depending on the specific cellular context and the status of the circadian rhythms [236]. Circadian factors like CRY1/2 and PER1 play marked roles in apoptosis regulation through various pathways. While PER2 enhances cancer cell sensitivity to radiation-induced apoptosis, PER1 knockdown increases apoptosis by altering the expression of antiapoptotic and proapoptotic genes. Conversely, CLOCK inhibits apoptosis, as evidenced by decreased expression of apoptosis-inducing factors in CLOCK-defective mice, leading to enhanced tumor growth [243,244]. This underscores the intricate involvement of circadian mechanisms in modulating cancer cell death.

We are beginning to understand the potential role of crosstalk between circadian rhythm machinery and aging in sustaining proliferative signals and evading apoptosis in cancers. Zhang et al. [245] found that the anticancer molecule MLN4924, a Nedd8-activating enzyme inhibitor, induces cell cycle arrest, apoptosis, and senescence in cancer cells. It suppresses osteosarcoma cell proliferation by causing G2/M arrest and apoptosis. It achieved this by stabilizing RORα and up-regulating its transcriptional targets, p21 and BMAL1. While p21 has a minimal role, BMAL1 suppression attenuates MLN4924’s antiproliferative effect, indicating that MLN4924-induced growth inhibition in osteosarcoma cells is mediated primarily by BMAL1. These findings underscore MLN4924 as a promising therapeutic for osteosarcoma treatment, implicating circadian rhythm components RORα and BMAL1 in its mechanism of action. In addition, Gul et al. [246]. identified a molecule, M47, that destabilizes CRY1, leading to an increase in circadian period length and enhanced apoptosis in certain cancer cells. M47 selectively enhances CRY1 degradation by increasing its ubiquitination, predominantly in the nucleus. M47-mediated CRY1 reduction enhances oxaliplatin-induced apoptosis in Ras-transformed p53-null fibroblast cells. Repeated M47 administration extends the median lifespan of p53^−/−^ mice by about 25%, indicating its potential as a treatment for cancers dependent on p53 mutation. Mechanistically, this significant lifespan extension suggests that M47 might influence aging-related processes, particularly in the context of an organism predisposed to cancer due to a p53 mutation. The extension of lifespan by M47 appears to be linked to its ability to enhance apoptosis in response to genotoxic stress, which could reduce the accumulation of damaged or potentially cancerous cells [243,246]. However, further studies still need to confirm whether M47 directly affects aging-related processes. Furthermore, Balounová et al. [247]. found that aging disrupts the rhythmicity of cell cycle genes in the colon, while tumorigenesis mainly affects circadian rhythms without altering their coupling with the cell cycle, highlighting different impacts of aging and cancer on circadian–cell cycle coordination.

### Aging, circadian rhythms, and genomic instability in cancer

Genomic instability can manifest as DNA damage, chromosomal instability, microsatellite instability, telomere dysfunction, replication errors, epigenetic changes, and impaired DNA repair and so on [8,168,248]. This instability may arise from various factors including errors in DNA replication, defects in DNA damage repair mechanisms, exposure to environmental carcinogens, and genetic predispositions, among others [249]. It is closely associated with the occurrence and progression of many diseases such as cancer, aging, and certain genetic disorders, among others. In order to prevent the replication of damaged DNA, organisms have evolved a sophisticated signaling network known as the DNA damage response (DDR) in eukaryotes [250]. Nevertheless, despite these mechanisms, not all DNA damage can be fully repaired, and their efficiency tends to diminish with age. The relentless buildup of genomic damage within cells consequently increases susceptibility to cancer and other age-related diseases [251]. In addition, there is a close interplay existing between the DDR and the circadian rhythm machinery. Numerous genes involved in DDR exhibit circadian rhythms in both mRNA expression and protein levels [252]. Circadian rhythm disruption can lead to dysregulation of DDR genes, which contributes to the hallmarks of cancer [253]. Mechanistically, some circadian rhythm components can directly interact with components of the DDR pathway. For example, upon DNA damage, PER1 interacts with ataxia telangiectasia mutated (ATM) protein and checkpoint kinase (Chk) 2, influencing ATM activity [254]. Reduced PER1 levels hinder Chk2 phosphorylation, affecting the response to DNA damage. Additionally, CRY1 and CRY2 regulate ATR activity by facilitating interactions with TIMELESS (TIM) and Chk 1, respectively [255].

Currently, there is limited direct evidence regarding the crosstalk between aging and circadian rhythms on the DDR pathway in tumor cells. Basic helix–loop–helix family member e40 (BHLHE40), also known as differentially expressed in chondrocytes 1 (DEC1) or stimulated by retinoic acid gene 13 protein(Stra13), may serve as a key molecular hub connecting these 3 entities [256,257]. On the one hand, BHLHE40 acts as a transcription factor to directly regulate the expression of core circadian rhythm genes. Similar to NR1D1, BHLHE40 is transactivated by CLOCK via E-box elements in their promoters. However, BHLHE40 represses its own transcription by binding directly to BMAL1 and competing with CLOCK for E-box occupancy [258–260]. On the other hand, BHLHE40 is widely utilized as an indicator of cellular senescence in vivo, and its capacity to induce cellular senescence has been documented in vitro as well [261,262]. BHLHE40 expression can be induced by p53 and DNA damage, and its overexpression promotes premature senescence, indicating its role as a mediator downstream of p53 in this process [263–265]. However, the role of BHLHE40 in tumors remains controversial, and its mechanisms may vary in different types of cancer. Several studies suggest that the expression level of BHLHE40 is closely associated with the occurrence and progression of various tumors. For instance, in certain types of cancer like gastric cancer [266], breast cancer [267], and colorectal cancer [268], the expression level of BHLHE40 is significantly increased. Additionally, BHLHE40 has been found to be correlated with tumor proliferation, invasion, and metastasis [266,269]. However, some studies also suggested that BHLHE40 may act as a protective factor in certain tumors. In esophageal squamous cell carcinoma, overexpressed BHLHE40 was found to be correlated with better survival and in vitro experiments demonstrated that overexpression of BHLHE40 induced cellular senescence and suppressed cell growth and colony formation in the esophageal squamous cell carcinoma cell line EC9706 [265]. Considering DNA damage repair, Ming et al. [270]. found that BHLHE40 could activate the promoter of the clusterin (CLU) gene, leading to increased secretory CLU expression, while BHLHE40 knockdown decreased secretory CLU expression upon DNA damage. Conversely, secretory CLU knockdown enhanced DNA damage-induced cell death in breast cancer cells, suggesting that BHLHE40 promoted cell survival by up-regulating secretory CLU to reduce the apoptotic response to DNA damage, providing insights into their roles in breast cancer progression.

Additionally, cell cycle and apoptosis regulator 2 (CCAR2) plays a pivotal role in connecting aging, circadian rhythms, and genomic instability in tumors. CCAR2 also known as deleted in breast cancer 1 (DBC1), has emerged as a pivotal regulator of transcriptional processes and multiple cellular processes [271,272]. It assumes diverse roles in both physiology and pathophysiology, serving as a regulator of DNA repair, cellular senescence, circadian rhythms, metabolism, tumorigenesis, and so on [271]. The protein CCAR2 undergoes direct phosphorylation by ATM, the primary kinase responsible for recognizing DNA damage. This phosphorylation event empowers CCAR2 to actively engage in orchestrating the cellular response to DNA damage, including processes such as DNA repair and apoptosis [273,274]. In addition, CCAR2 suppresses carboxy-terminal interacting protein-mediated double-strand break (DSB) end resection and homologous recombination repair, while simultaneously facilitating the p53 binding protein 1 (53BP1)–Rap1-interacting factor (RIF1)–Shieldin pathway to stimulate nonhomologous end joining (NHEJ) repair [275]. Moreover, CCAR2 was also found to be involved in DNA damage-induced cellular senescence. Specifically, when 3T3-L1 preadipocytes are treated with H_2_O_2_, there is a rapid increase in CCAR2 binding to and inhibition of HDAC3. This inhibition ultimately leads to the induction of expression of 2 important upstream elements of the senescence program, p16INK4a and p21waf1 [276]. Importantly, there were studies suggesting that CCAR2 played a role in modulating circadian rhythms by interacting with and enhancing the stability and repressive function of the NR1D1. CCAR2-mediated repression of BMAL1, a transcriptional activator, is also facilitated through NR1D1. Additionally, CCAR2 interacts with proteins within the PER complex, involved in circadian gene regulation, and modulates the expression of CLOCK and BMAL1 transcription factors, thereby influencing circadian oscillations [277,278]. These results highlight the multifaceted role of CCAR2 in regulating circadian rhythm, potentially through interactions with various protein complexes involved in circadian gene expression.

### Cellular senescence, circadian rhythms, and cancer

Cellular senescence is marked by permanent growth arrest in response to factors like telomere dysfunction, oncogene activation, and persistent DNA damage [279,280]. This process typically involves a decline in cellular metabolism, proliferation, and repair capabilities, as well as the deterioration of internal cellular structures and functions. Cellular senescence is an important aspect of organic aging and is influenced by various factors including genetics, environment, lifestyle, and intracellular biochemical processes [281,282]. Senescent cells exhibit morphological and metabolic alterations, chromatin remodeling, changes in gene expression, and develop a pro-inflammatory phenotype known as the SASP [279]. The biological effects of cellular senescence are multifaceted, with senescent cells demonstrating both protective and deleterious roles, largely contingent on the physiological milieu. Although senescence may have evolved as a mechanism to prevent the malignant transformation of damaged cells, its occurrence can contribute to various age-related pathologies, such as cancer, tissue degeneration, and inflammatory diseases [283,284]. A recent research suggests that pharmacological activation of circadian rhythms suppressors can impact the survival capability of cancer cells by inhibiting pathways that are aberrantly activated in cancer and lead to specific death of OIS cells [285]. Sulli et al. [285]. found that SR9009 and SR9011, which were 2 different agonists of NR1D, could selectively induce cell death in cancer cells and OIS cells without harming normal cells. These agonists exhibit anticancer effects across various oncogenic drivers, independent of p53 and under hypoxic conditions, by regulating autophagy and de novo lipogenesis. Importantly, NR1D agonists inhibit glioblastoma growth in vivo and improve survival in mice without causing significant toxicity, suggesting their potential as effective and safe anticancer agents with a broad therapeutic window [285]. In addition, circadian rhythm components appear to play unique roles in tumor drug resistance and therapy-induced senescence (TIS). TIS refers to the phenomenon that cancer cells undergo senescence in response to treatments such as ionizing radiation or chemotherapy [286]. Jia et al. [287]. found that bladder cancer cells resistant to cisplatin did not exhibit apparent senescence upon treatment with paclitaxel (PTX), unlike the nonresistant cells. Instead, the resistant cells entered a quiescent state characterized by prolonged circadian rhythms. This quiescent state was associated with the accumulation of the circadian protein CRY1. Knockdown of CRY1 restored PTX-induced senescence and mechanistically CRY1 prevented senescence by promoting degradation of the tumor suppressor protein p53, potentially through increased binding of p53 to its ubiquitin E3 ligase MDM2 proto-oncogene. Furthermore, immune cell senescence has also been found to be closely associated with the tumorigenesis and progression of tumors. Zeng et al. [288]. investigated the mechanism underlying the impairment of NK cell immunosurveillance induced by chronic circadian disruption. Mice exposed to light–dark reverse every 4 days for 12 weeks to disrupt normal circadian rhythm exhibited suppressed mRNA and protein levels of PER1 and PER2, along with increased expression of CLOCK, indicating successful generation of a circadian rhythm disruption model. Chronic circadian disruption led to NK cell aging, characterized by reduced expression of Ly49 family receptors. Additionally, chronic shift-lag inhibited NK cell secretion of granular CD107a and interferon gamma, impaired clearance of MHC-I-deficient tumor cells by NK cells, and promoted lung metastasis of B16F10 melanomas. These effects were attributed to reduced NK cell killing function, possibly mediated by decreased expression of the Eomes transcription factor, which, in turn, suppresses CD122 transcription. Overall, these findings suggested that chronic circadian disruption attenuated NK cell cytolytic activity by promoting NK cell senescence and down-regulating CD122 expression.

### Aging, circadian rhythms, and deregulated cellular metabolism in cancer

Disrupted nutrient sensing is widely recognized as a hallmark of aging as evidenced by existing research [7]. Additionally, the reprogramming of energy metabolism has been integrated into the established hallmarks of cancer [8]. In fact, there is a close relationship between the circadian rhythms and cellular metabolism. The circadian rhythm system can regulate the activity of many metabolic pathways within cells, leading to different metabolic characteristics at different times of the day [289,290]. However, the crosstalk and molecular mechanisms among aging, circadian rhythms, and deregulated cellular metabolism in cancer remain largely unknown. Some important molecules and pathways may serve as key mediators linking them together.

AMPK and Sirtuins: Sirtuins (SIRTs) are a family of 7 NAD^+^-dependent proteins involved in cellular processes such as gene regulation, DNA repair, metabolism, and aging [289,291]. SIRT1, the most studied sirtuin, requires NAD^+^ as a cofactor for its enzymatic activity. Both AMP-activated protein kinase (AMPK) and SIRT1 are key regulators in cellular responses to low-energy states [2]. AMPK is a master regulator of cellular energy homeostasis. It becomes activated in response to an increase in the AMP:ATP ratio, signifying low energy levels within the cell. Upon activation, AMPK triggers several pathways to restore energy balance, such as promoting glucose uptake and fatty acid oxidation while inhibiting energy-consuming processes like protein synthesis [2,292]. SIRT1, on the other hand, is also influenced by cellular energy status, particularly through its dependence on the NAD^+^/NADH ratio [293]. The interaction between AMPK and SIRT1 forms a positive feedback loop. AMPK activation can increase the levels of NAD^+^ through its downstream effects, leading to the activation of SIRT1. Conversely, activated SIRT1 can deacetylate and activate proteins that directly or indirectly regulate AMPK, thus amplifying the cellular response to low-energy states [2,294]. Overall, AMPK and SIRTs have been associated with promoting healthy aging and protecting against carcinogenesis. Activation or up-regulation of these proteins has been shown to inhibit pathways involved in cancer cell growth and stemness maintenance. For instance, activated AMPK can phosphorylate and inhibit key components of pathways crucial for cancer cell proliferation and survival [2,292]. Moreover, SIRT1 expression is regulated by CLOCK/BMAL. SIRT1 interacts directly with CLOCK, impacting its acetylation status. Additionally, the interaction between SIRT1 and PER2 affects circadian rhythms through PER2 deacetylation [295–297]. SIRT3 influences enzymes involved in the tricarboxylic acid cycle, potentially connecting mitochondrial metabolism with circadian rhythms. Moreover, SIRT6 governs the recruitment of Sterol-regulatory element binding protein 1, a key player in fatty acid metabolism, by the CLOCK/BMAL complex to circadian promoters, thus intertwining circadian rhythms with lipid synthesis and metabolism [297–299].

mTOR: The mTOR (mechanistic target of rapamycin) kinase is part of the multiprotein complexes mTORC1 and mTORC2, which serves as an intracellular nutrient sensor, indicating a high cellular energy state. Its expression is up-regulated during senescence, and increased mTOR activity is recognized as a characteristic of aging [300,301]. Genetically modified mice with reduced mTORC1 activity exhibit extended lifespan, indicating the role of mTOR in longevity regulation [302]. Increased mTOR activity in aging hypothalamic neurons contributes to age-related obesity, a significant cancer risk factor, while mTOR activation in cancer cells promotes tumor growth and metabolic reprogramming, highlighting its diverse roles in health and disease [7,303]. Given the circadian rhythms, mTOR activity has been observed to exhibit rhythmicity, aligning with patterns of food intake, and this phenomenon can occur independently from the light-dependent circadian mechanism [304]. In addition, BMAL1 is identified as a substrate of the mTOR effector kinase S6K1. Phosphorylation of BMAL1 by S6K1 is crucial for its interaction with the translation machinery, facilitating rhythmic protein synthesis [305]. Furthermore, PER2 acts as a scaffold protein to inhibit the activity of the mTORC1 complex. Loss of PER2 results in heightened protein synthesis and increased cell proliferation [306].

**Fig. 4.**
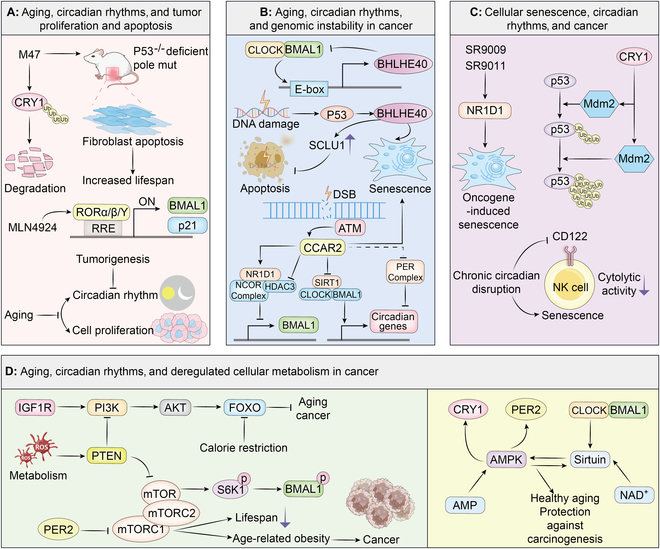
The common hallmarks and underlying mechanisms among cancer, circadian rhythms, and aging. (A) Aging, circadian rhythms, and tumor proliferation and apoptosis. M47 is a molecule, which can destabilize CRY1, increasing circadian period length and enhancing apoptosis in certain cancer cells, particularly by promoting CRY1 degradation through ubiquitination in the nucleus. This reduction in CRY1 also amplifies oxaliplatin-induced apoptosis in Ras-transformed p53-null fibroblasts and extends the median lifespan of p53^−/−^ mice by about 25%, indicating its potential as a cancer treatment targeting p53 mutations. MLN4924 can trigger cell cycle arrest, apoptosis, and cellular senescence in cancer cells. It achieves this by stabilizing RORα and up-regulating its transcriptional targets, p21 and BMAL1. (B) Aging, circadian rhythms, and genomic instability in cancer. BHLHE40 and CCAR2 are 2 key molecular hubs connecting aging, circadian rhythms, and genomic instability in cancer. On the one hand, BHLHE40 acts as a transcription factor that directly regulates the expression of core circadian genes. BHLHE40 is transactivated by the CLOCK:BMAL1 through the E-box element in its promoter. Instead, BHLHE40 represses its own transcription by directly binding to the BMAL1 protein and competing with CLOCK:BMAL1 for the occupancy of E-box sequences in its promoter. In addition, BHLHE40 expression can be induced by p53 and DNA damage, and its overexpression promotes premature senescence, indicating its role as a mediator downstream of p53 in this process. Moreover, BHLHE40 can promote cancer cell survival by up-regulating SCLU to reduce the apoptotic response to DNA damage. CCAR2 is found to be involved in nonhomologous end joining (NHEJ) repair and DNA damage-induced cellular senescence. Moreover, CCAR2 modulates circadian rhythms by interacting with and stabilizing the NR1D1, enhancing its repressive function. CCAR2 also represses the transcriptional activator BMAL1 via NR1D1 and interacts with proteins in the PER complex, thereby influencing the expression of CLOCK and BMAL1 and affecting circadian oscillations. (C) SR9009 and SR9011 are 2 distinct NR1D1 agonists that selectively induce cancer cell- and oncogene-induced senescent cell death. In addition, CRY1 prevented senescence by promoting degradation of the tumor suppressor protein p53, potentially through increased binding of p53 to its ubiquitin E3 ligase MDM2 proto-oncogene. Importantly, chronic circadian disruption attenuates the cytolytic activity of NK cells by promoting NK cell senescence and down-regulating the expression of CD122. (D) Aging, circadian rhythms, and deregulated cellular metabolism in cancer. Aging, circadian rhythms, and tumor metabolism are connected by 3 key pathways, IIS axis, mTOR pathway, and AMPK pathway, and the sirtuin family of deacetylases. CRY, cryptochrome; RORα, recombinant receptor tyrosine kinase like orphan receptor α; RRE, NR1D/ROR response elements; BMAL1, brain and muscle arnt-like 1; CLOCK, circadian locomotor output cycles kaput; BHLHB40, basic helix–loop–helix family member e40; SCLU, secretory clusterin; DSB, DNA double-strand break; ATM, ataxia-telangiectasia mutated; CCAR2, cell cycle and apoptosis regulator 2; PER, period; HDAC3, histone deacetylase 3; NCOR, nuclear receptor co-repressor; Mdm2, mouse double minute 2 homolog; IGF1R, insulin-like growth factor 1 receptor; PI3K, phosphatidylinositol 3-kinase; ATK, protein kinase B; FOXO, forkhead box O; PTEN, phosphatase and tensin homolog deleted on chromosome ten; mTOR, mechanistic target of rapamycin; S6K1, ribosomal protein S6 kinase beta-1; AMPK, AMP-activated protein kinase; NAD, nicotinamide adenine dinucleotide.

Insulin and IGF-1 signaling axis: The insulin/IGF-1 signaling (IIS) pathway serves as a mechanism through which cells sense glucose levels, making it the most evolutionarily conserved pathway for regulating aging processes [2,307]. Insulin/IGF1 bind to their respective receptors, activating the IIS signaling pathway and triggering a kinase cascade that activates AKT. AKT then phosphorylates FOXO, inhibiting its transcriptional activity and promoting cell survival, growth, and proliferation [308,309]. Additionally, the IIS signaling pathway interacts with pathways such as mTOR and AMPK, forming a complex regulatory network governing lifespan and aging [308,310]. The IIS pathway plays a vital role in regulating cellular processes and impacts both longevity and cancer susceptibility. FOXO transcription factors, downstream effectors of the IIS pathway, are implicated in mediating the beneficial effects of caloric restriction on aging and exert tumor-suppressive functions [311,312]. Modulating IIS activity through interventions such as inhibition of IGF1R extends lifespan and improves healthspan in animal models, while also enhancing anticancer immunosurveillance [2,313,314]. Targeting the IIS axis represents a promising avenue for antiaging and antitumor strategies, offering potential therapeutic interventions to promote healthy aging and combat cancer progression. Given the circadian rhythms, oxidative stress disrupts the PI3K/AKT signaling pathway by inhibiting PTEN function, leading to increased activation of AKT. This disruption activates BMAL1 in an mTOR-dependent manner, highlighting a complex interplay between oxidative stress, PTEN, PI3K/AKT signaling, BMAL1, and mTOR in cellular responses to oxidative stress and potentially in the regulation of circadian rhythms [315]. Figure 4 summarizes the common hallmarks and underlying mechanisms among cancer, circadian rhythms, and aging.

## Perspective

### The complexity of circadian rhythms in regulating aging and cancer

Circadian rhythm genes hold an upstream position in the gene regulatory network. They influence the expression of thousands of genes, including those involved in cellular senescence [2]. This regulation is mediated through the core TTFL and associated transcription factor networks [2,24]. These genes exhibit a 24-h expression cycle across different tissues and organs and are subject to epigenetic regulation and modulation by ncRNAs. Through these mechanisms, circadian rhythm genes coordinate the physiological functions of the organism, ensuring its adaptation to the external environmental day–night changes [9,316]. In the tumor state, circadian rhythm genes significantly influence cancer development and progression through various mechanisms, including the regulation of the cell cycle, DNA repair, metabolic pathways, immune function, oxidative stress response, and the tumor microenvironment [237]. Disruption or abnormal expression of these genes can lead to uncontrolled cell proliferation, accumulation of DNA damage, metabolic imbalances, and weakened immune surveillance, thereby increasing cancer risk and promoting tumor growth [237]. Therefore, due to the involvement of numerous mechanisms, the regulation of tumor development by circadian rhythm genes is multifaceted, with cellular senescence being just one aspect of this complex process. This suggests that even if 2 genes have potentially opposite effects on cellular senescence, it does not necessarily mean that they will have opposite oncological roles (tumor-suppressing or tumor-promoting) in a specific type of tumor. Specifically, for example, BMAL1 plays an important role in cellular senescence. Research indicates that BMAL1 helps maintain genomic stability, suppresses the activation of transposable elements like LINE1, and combats cellular senescence. The absence of BMAL1 can lead to genomic instability and increased oxidative stress, accelerating cellular senescence [229]. Additionally, BMAL1 influences the aging process by modulating the mTOR signaling pathway. Increased mTORC1 activity has been linked to BMAL1 deficiency, highlighting an important role of BMAL1 in regulating cellular metabolism and senescence [317]. Therefore, from the perspective of cellular senescence, reduced expression of BMAL1 in breast cancer may weaken antisenescence defense mechanisms, making cancer cells more prone to accumulating DNA damage and promoting cancer progression. However, there was also a study indicating that BMAL1 could promote breast cancer cell invasion and metastasis by up-regulating MMP9 expression through the activation of the NF-κB signaling pathway [38]. Similarly, the role of CRY2 in cellular senescence is a complex and multifaceted topic. Although there is currently no direct research specifically exploring the connection between CRY2 and cellular senescence, some indirect evidence provides insights into its potential role. For instance, CRY2 can regulate the G1/S phase transition of the myoblast cell cycle by stabilizing Cyclin D1 mRNA. This regulation is essential for maintaining normal cell proliferation and differentiation. Cells lacking CRY2 tend to exit the cell cycle prematurely, leading to reduced cell proliferation efficiency [318]. In addition, research has found that in CRY2-deficient cells, the introduction of oncogenes leads to increased expression of ATF4 [226]. ATF4 is a potent suppressor of proteins associated with cellular senescence, such as p16INK4a and p19ARF [319,320]. The up-regulation of ATF4 inhibits the expression of these proteins, thereby preventing cellular senescence. Moreover, research suggests that certain CRY2 mutations could inhibit P53, thereby enhancing cell proliferation [321]. P53 responds to various cellular stress signals, such as DNA damage, telomere shortening, and oxidative stress, by activating downstream target genes like p21, leading to cell cycle arrest and the induction of cellular senescence [322]. From the perspective of cellular senescence, CRY2 may promote cellular senescence by inhibiting the up-regulation of ATF4, which could prevent carcinogenesis. Conversely, it might also suppress cellular senescence through inhibition of p53, potentially contributing to cancer progression. These findings suggest that CRY2’s role could be context-dependent, with its effects varying depending on the biological environment and disease stage. However, direct experimental evidence supporting these dual roles of CRY2 in cellular senescence and cancer progression is still limited. Further studies are needed to clarify the underlying mechanisms and to confirm these potential pathways in different cellular contexts. In summary, these findings illustrate the complex regulatory network of circadian rhythms in controlling cellular senescence and tumorigenesis. They highlight the intrinsic potential of future anticancer therapies based on restoring circadian function. However, the lack of direct studies specifically connecting CRY2 to cellular senescence represents an important gap in our current understanding and warrants further investigation.

Additionally, circadian rhythms may serve as an important intermediary process or factor in the tumorigenesis and progression of cancer and aging. On the one hand, circadian rhythms, through the regulation of inflammatory factors like SASP or key hormones within the endocrine system, such as melatonin, cortisol, IGF-1, and growth hormone, may play a crucial bridging role in the interconnected processes of aging and tumorigenesis [323–325]. As individuals age, disruptions in circadian rhythms can lead to altered secretion patterns of hormones, such as decreased melatonin, dysregulated cortisol rhythms, and impaired IGF-1 signaling pathways [325–327]. These hormonal changes not only accelerate cellular aging and functional decline in tissues but also potentially trigger chronic inflammatory responses and weaken the immune system, thereby creating a favorable environment for tumor development and progression. For example, the reduction of melatonin in aging is associated with decreased immune function and increased oxidative stress, while its diminished antitumor effects may facilitate the growth and metastasis of cancer cells [328,329]. Similarly, abnormal elevations in cortisol may exacerbate neurodegenerative processes and immune system aging through chronic stress mechanisms, simultaneously promoting cancer cell proliferation and spread [324,330]. Therefore, the intricate relationship between circadian rhythms and the endocrine system forms a network of interactions influencing both aging and cancer development. Future research aimed at elucidating these mechanisms could facilitate the development of circadian rhythm-based interventions that not only delay the aging process but also reduce cancer incidence, offering new strategies for the health management of the elderly population.

### Focus attention on the hallmarks of cancer and aging

Although many epidemiological studies have demonstrated correlations among circadian rhythms, aging and cancer, the underlying molecular mechanisms remain largely unclear. The majority of existing research has focused on cellular senescence, cellular metabolism and genomic instability. However, it has been reported that there are 14 hallmarks of cancer and 12 hallmarks of aging [7,8]. Numerous other important hallmarks have yet to receive attention. For example, tumor-promoting inflammation is an important hallmark of cancer. Chronic inflammation within the tumor microenvironment can fuel cancer progression by promoting cell proliferation, angiogenesis, and tissue remodeling. Inflammatory cells and cytokines play key roles in this process [331]. Clinically, targeting the inflammatory components within the tumor microenvironment has emerged as a promising therapeutic approach. The use of anti-inflammatory agents, such as nonsteroidal anti-inflammatory drugs or selective COX-2 inhibitors, has shown potential in reducing tumor-associated inflammation and preventing tumorigenesis [332,333]. Furthermore, cytokine inhibitors, such as interleukin-6 (IL-6) or tumor necrosis factor-α (TNF-α) blockers, are being explored for their ability to disrupt the pro-tumorigenic signaling pathways driven by chronic inflammation. This strategy aims not only to slow down tumor growth but also to improve the efficacy of standard therapies like chemotherapy and immunotherapy by modulating the tumor microenvironment [334–336]. Additionally, inflammation-related biomarkers, including C-reactive protein and serum cytokine levels, are under investigation for their potential to predict treatment responses and guide personalized treatment plans [337,338]. These approaches underscore the importance of inflammation as a therapeutic target in cancer treatment, with the goal of improving overall patient outcomes. With aging, the immune system undergoes changes, including a phenomenon called immunosenescence, where the immune response becomes less efficient and may result in chronic activation of inflammatory pathways. Moreover, the persistent presence of senescent cells contributes to chronic inflammation, exacerbating inflammaging by releasing an excess of pro-inflammatory cytokines typical of the SASP [339]. Notably, research related to circadian rhythms suggests that the immune system also exhibits noticeable rhythmicity. The activity of the immune system varies at different times of the day, which may affect the occurrence and severity of inflammation. For instance, studies indicate that inflammatory responses may vary throughout the day, with certain periods being more conducive to inflammation while others may be more attenuated. Additionally, the polymorphic microbiome has garnered more and more attention in recent years. The polymorphic microbiome refers to a microbial community that exhibits a high degree of diversity and variability in its composition and function [2,8,340]. This polymorphism can occur due to genetic variation among microbial species, differences in environmental factors, and host-specific factors such as diet, immune response, and overall health [341,342]. Some studies suggest that as individuals age, there are changes in the microbiome [343]. These changes may be associated with declining immune function, alterations in metabolism, and increased incidence of chronic diseases [344,345]. Compared to healthy young individuals, older adults exhibit greater microbial diversity and differences in microbial composition, possibly linked to the declining function of the immune and digestive systems during aging [346,347]. Circadian rhythms, like the sleep–wake cycle and meal patterns, profoundly influence the composition and function of the gut microbiota, while the gut microbiota reciprocally impacts circadian rhythms. Irregular eating habits or disrupted sleep patterns can alter gut microbial balance, potentially leading to health issues. Conversely, gut microbes produce metabolites and neurotransmitters that influence host circadian rhythms, emphasizing the bidirectional relationship [348,349]. Furthermore, the relationship between tumors and the microbiome is intricate. Changes in the microbiota have been associated with tumor initiation and progression. Microbes can influence tumorigenesis by modulating host immune responses, activating oncogenes, or producing carcinogenic substances. Additionally, the microbiome plays a crucial role in tumor therapy response, with specific microbial components influencing the efficacy of immunotherapy, suggesting the potential for microbiome-based interventions to enhance treatment outcomes [350,351]. For example, prostate cancer is typically considered a “cold” tumor, meaning that immune checkpoint inhibitors are generally less effective against it. A study showed that using patient-derived prostate-specific microbiome CP1 in combination with anti-PD-1 immunotherapy increased survival rates and reduced tumor burden [352,353]. In summary, the microbiome exerts complex effects on aging, circadian rhythms, and tumors, involving the regulation and development of various physiological and pathological processes. Future research will further elucidate the mechanisms by which the microbiome influences these processes, providing new insights and approaches for the prevention and treatment of related diseases.

### Chronotherapy, key to clinical practice

In addition to the need to further explore the mechanism, translating existing research findings into clinical practice is also an area that requires considerable effort.

Chronotherapy, the strategic timing of medication administration in accordance with the body’s circadian rhythms, holds promise for optimizing cancer treatment efficacy while minimizing side effects [20,354,355]. Cancer cells and normal cells exhibit significant differences in their circadian rhythms. Normal cells typically follow a more stable circadian rhythm, while cancer cells often display disrupted or aberrant rhythmic patterns. This discrepancy forms the basis of chronotherapy, as it allows for treatments to be scheduled at times when cancer cells are most vulnerable and normal cells are best able to repair and recover [20,316,356,357]. Specifically, the molecular mechanisms underlying chronotherapy are intricately linked to the circadian regulation of various cellular processes, including cell cycle progression, DNA repair, apoptosis, and metabolism. The circadian rhythms control the timing of the cell cycle, which is crucial for tumor growth and the effectiveness of chemotherapy. The expression of CDKs and their inhibitors is regulated by core clock genes, such as BMAL1, CLOCK, PERs, and CRYs. This regulation creates a temporal window during which tumor cells are more susceptible to DNA-damaging agents. For example, studies have shown that the administration of the chemotherapeutic agent oxaliplatin in sync with the peak expression of DNA repair genes can minimize damage to healthy tissues while maximizing toxicity to cancer cells [358,359]. This is due to the fact that DNA repair mechanisms are less active in tumor cells at specific times of the day, making them more vulnerable to treatment. In addition, the timing of treatment can also influence the apoptotic pathways and DNA repair mechanisms within cancer cells. The circadian rhythms regulate the expression of key proteins in these pathways, such as p53, BAX, BCL-2, and ATM, which are involved in the response to DNA damage induced by chemotherapy and radiotherapy. For instance, during radiation-induced DNA DSBs, PER1 binds to ATM to halt cell cycle progression and triggers p53-mediated apoptosis if the damage persists [360]. Ectopic expression of PER1 impairs malignant growth, and reduced levels of endogenous PER1 are found in human breast cancer [92]. Additionally, PER2 acts as a tumor suppressor and is essential in DDR. In murine models, it is required for radiation-induced up-regulation of clock gene proteins, enhancing tumor suppression and survival [361]. In human cells, both PER1 and PER2 facilitate apoptotic pathways driven by the tumor suppressor protein p53 [19,360,362]. This suggests that radiation efficacy could potentially be enhanced during periods of high PER levels, which could be predicted based on a patient’s circadian phase or induced by manipulating environmental factors such as food intake. Moreover, the circadian rhythms also regulate the expression of enzymes involved in drug metabolism, such as cytochrome P450 enzymes, which are crucial for the activation and detoxification of chemotherapeutic agents. Disruptions in circadian rhythms can alter the pharmacokinetics of these drugs, leading to variations in their efficacy and toxicity. By carefully timing chemotherapy to coincide with these rhythms, chronotherapy can maximize the anticancer effects and reduce harm to healthy cells. Research has shown that this approach can significantly improve treatment responses and decrease side effects such as nausea, vomiting, hair loss, and bone marrow suppression [20,363,364].

Notably, in recent years, other related research of chronotherapy including chrono-radiotherapy, chrono-immunotherapy, and chrono-targeted therapy are also being gradually carried out [365–369]. A recent study published in *Cell* in 2024 indicated that the quality and quantity of tumor-infiltrating lymphocytes, particularly CD8^+^ T cells, were circadian rhythm dependent. Researchers found that the number of tumor-infiltrating lymphocytes significantly varies at different times of the day, peaking at night. Based on this phenomenon, they further discovered that both chimeric antigen receptor T (CAR T) cell therapy and anti-PD-1 therapy exhibit time dependency. Treatments administered at night are more effective than those given in the morning, highlighting the critical impact of treatment timing on the efficacy of immunotherapy [370]. Their results highlighted the functional significance of circadian dynamics in the tumor microenvironment and proposes that leveraging these rhythms can optimize immunotherapy effectiveness, paving the way for more personalized and effective cancer treatments. Similarly, myeloid-derived suppressor cells (MDSCs) also exhibit circadian rhythm dependency. Clock-regulated pro-inflammatory key cytokines modulate PD-L1-positive MDSCs, causing their numbers to fluctuate rhythmically in colorectal cancer. This rhythmic variation affects the efficacy of immunotherapy. By understanding these rhythmic changes and administering PD-L1 inhibitors when MDSCs are most abundant, optimal anticancer efficacy can be achieved [371]. Interestingly, sleep disorders disrupt circadian rhythms, leading to metabolic abnormalities that play a crucial role in promoting malignant tumor progression. Peng et al. [372] found that circadian rhythm disruption can affect the clock gene *CLOCK*, leading to its overactivation of long-chain acyl-CoA synthetase 1 (ACSL1), which catalyzes the synthesis of more palmitoyl-CoA (PA-CoA). PA-CoA, in turn, mediates the palmitoylation of the *CLOCK* gene, creating a positive feedback loop. This process prevents the degradation of CLOCK protein via the ubiquitin–proteasome pathway, thereby sustaining the enhanced stemness of cancer cells induced by circadian rhythm disruption, and continuously driving cancer development. Moreover, they found that the endogenous circadian rhythm-related hormone β-endorphin can mediate the down-regulation of CLOCK and ACSL1 expression. This effectively reverses the pro-cancer effects and enhanced stemness of cancer cells caused by sleep deprivation. Based on the sleep status of cancer patients, rhythmic supplementation of β-endorphin (similar to chronochemotherapy) may be an effective anticancer strategy. In summary, ongoing research efforts aim to further elucidate the molecular mechanisms underlying circadian-based interventions and expand the application of chronotherapy to other age-related conditions, promoting the integration of circadian principles into clinical practice for improved patient outcomes [370,373]. Table 1 summarizes clinical studies on chronotherapy in oncology [365–368,374–382]. In the future, further elucidating the potential relationship between the expression patterns of circadian rhythm molecules and the outcomes of chronotherapy holds significant importance in cancer treatment. Understanding this connection could enhance the effectiveness of therapeutic strategies by optimizing treatment timing based on the biological rhythms of both the patient and the tumor. On the one hand, future research could focus on integrating multi-omics sequencing technologies, including single-cell sequencing, and in vivo imaging techniques with clinical treatment efficacy assessments to better investigate the intrinsic biological mechanisms underlying chronotherapy and circadian factors [383]. These advanced methodologies will enable a deeper understanding of these complex biological processes and offer new insights for the development of personalized therapeutic strategies. On the other hand, individual circadian rhythms vary from person to person, influenced by factors such as genetics, lifestyle, and environmental conditions. The degree and nature of circadian disruption in tumor cells can also differ among patients, depending on their specific circumstances. By investigating the expression patterns of circadian molecules, these disruptions can be identified and used as biomarkers for circadian rhythm disturbances within tumors. Therefore, personalized chronotherapy can be designed based on individual specific circadian rhythm profile. By monitoring the expression patterns of circadian rhythm molecules in a patient, it is possible to identify the optimal timing for drug administration or other therapeutic interventions. This approach aims to enhance the efficacy of treatments while minimizing potential side effects.

**Table 1.** The summary of clinical study on chronotherapy in oncology

Author	Chronotherapy type	Number of patients	Tumor type	Treatment	Main results	Reference
Bjarnason et al.	Chrono-radiotherapy	205 patients	Head and neck cancer	50 Gy in 25 fractions vs. 60 Gy in 25 or 30 fractions vs. 66 Gy in 33 fractions vs. 70 Gy in 35 fractions	Morning irradiation (8–10 AM) showed better normal tissue tolerance and less weight loss than PM radiotherapy (4–6 PM) (52.9% vs. 62.4% grade 3 toxicity).	[374]
Goyal et al.	Chrono-radiotherapy	212 patients	Head and neck cancer	Be treated everyday with no more than one fraction per day, 2 Gy/fraction, 5 fractions per week without any intended gaps. and a planned target dose of 60 Gy	The grades of mucositis were marginally higher in the evening-irradiated group than in the morning-irradiated group, 38% vs. 26% (*P* = 0.08).	[365]
Zhang et al.	Chrono-chemotherapy	148 patients	Head and neck cancer	Two cycles of induction chemotherapy followed by 2 cycles of concurrent chemotherapy with intensity-modulated radiation therapy. Docetaxel (75 mg/m^2^) and cisplatin (75 mg/m^2^) were administered on day 1 through an intravenous drip, while 5-fluorouracil (750 mg/m^2^) was administered by a constant intravenous pump from day 1 to day 5 (120 h). One cycle of induction chemotherapy was 21 days	Cisplatin infusion peaking at 4 PM, compared to a constant rate infusion, significantly reduced the incidence of adverse effects: oral mucositis occurred in 73.9% vs. 87.7%, vomiting in 47.9% vs. 71.2%, and nausea in 66.7% vs. 79.5%.	[375]
Gou et al.	Chrono-chemotherapy	60 patients	Nasopharyngeal carcinoma	80 mg/m^2^ of cisplatin from 1000 to 2200 h; 1000 mg/m^2^ of 5-FU and 200 mg/m^2^ of citrovorum factor from 2200 to 1000 h for 3 days	Compared to constant administration, chronomodulation of treatment significantly reduced leukocytopenia, thrombocytopenia, and nausea/vomiting.	[376]
Lin et al.	Chrono-chemotherapy	124 patients	Nasopharyngeal carcinoma	DDP administration from 1000 to 2200 h; 5-FU administration from 2200 to 1000 h	Compared to constant administration, significant decrease in stomatitis during radiotherapy.	[377]
Lévi et al.	Chrono-chemotherapy	92 patients	Colorectal cancer	Daily administration of 5-FU (600 mg/m^2^ per day), FA (300 mg/m^2^ per day), and 1-OHP (20 mg/m^2^ per day) for 5 days and were repeated every 21 days (16-day intermission) in ambulatory patients with the use of a programmable in-time pump	Objective response rate higher in the chrono arm: 53% vs. 32%, *P* = 0.038; median survival higher in the chrono arm: 19 vs. 14.9 months (*P* = 0.03). Rate of severe stomatitis higher in conventional arm: 89% vs. 18%, *P* < 0.001.	[378]
Giacchetti et al.	Chrono-chemotherapy	564 patients	Metastatic colorectal cancer	The first course: FU (3,000 mg/m^2^), LV (1,200 mg/m^2^), and oxaliplatin (100 mg/m^2^). An escalation of FU by 400 mg/m^2^/course on the second course and by 200 mg/m^2^ on the third course was planned if no grade 2 toxicity had occurred. In patients with grade 2 toxicity, the doses remained unchanged	Main acute toxicities were neutropenia (3-fold higher rate in the conventional arm) and diarrhea (3-fold lower frequency in the conventional arm). Other gastro/skin toxicities were more frequent in the chronomodulated arm.	[379]
Shukla et al.	Chrono-radiotherapy	229 patients	Cervical cancer	A regimen of external radiation to the whole pelvis using anterior and posterior parallel opposing fields to a dose of 50 Gy in 25 fractions at 5 fractions/week, using a Cobalt 60 teletherapy unit followed by intracavitary brachytherapy	Diarrhea, both overall (grade I–IV) and higher grade (III and IV), was significantly more common in the morning treatment arm compared to the evening arm (overall: 87.39% vs. 68.18%, *P* < 0.01; higher grade: 14.29% vs 5.45%, *P* < 0.05). Other radiation-induced toxicities were higher in the morning arm but not significantly different, and posttreatment radiation responses were similar between both groups.	[366]
Chang et al.	Chrono-radiotherapy	67 patients	Cervical cancer	External beam radiotherapy (RT) (50 Gy in 25 fractions) and brachytherapy (36–42 Gy in 6–7 fractions) twice every week	Radiation-induced mucositis occurred significantly more frequently in the morning group, both overall (75% vs. 57.6%) and in high-grade cases (12.5% vs. 6.1%) (*P* < 0.05). There were no significant differences in tumor control between the groups. However, hematologic toxicities were reduced in the morning group.	[380]
Li et al.	Chrono-chemotherapy	41 patients	Advanced non-small cell lung cancer	75 mg/m^2^ of docetaxel on day 1; 20 mg/m^2^ of cisplatin on days 1–4 at either 0600 h or 1800 h; 1,000 mg/m^2^ of gemcitabine on days 1 and 8	No significant difference in total response rate between the chronotherapy group (52.94%) and the routine chemotherapy group (50.00%), *P* = 0.853. The rates of nausea, neutropenia, and leucopenia were lower in the chronotherapy group.	[381]
Damato et al.	Chrono-chemotherapy	166 patients	Glioblastoma	Temozolomide in the morning or evening	Patients taking morning temozolomide exhibited longer overall survival (OS) compared to evening (median OS, 95% confidence interval [CI] = 1.43, 1.12–1.92 vs. 1.13, 0.84–1.58 years).	[382]
Escudier et al.	Additional chronotherapies	107 patients	Metastatic renal cell carcinoma	Sunitinib: at a starting dose of 37.5 mg. The dosage was titrated up to 50 mg/d or down to 25 mg/d depending on tolerability	Time of day had no significant effect on efficacy, tolerance, or quality of life.	[367]
George et al.	Additional chronotherapies	60 patients	Gastrointestinal stromal tumor	Morning or evening dosing of sunitinib 37.5 mg/d	Time of day had no significant effect on efficacy or adverse events.	[368]

### Targeting senescence: Emerging cancer therapies and future directions

Moreover, in recent years, as our understanding of how aging affects tumorigenesis and tumor progression has deepened, leveraging and intervening in aging has emerged as a novel field in cancer therapy. Immunosenescence refers to the gradual deterioration of the immune system associated with aging. This process leads to increased susceptibility to infections, cancer, and autoimmune diseases in the elderly, as well as reduced efficacy of vaccines [384,385]. Two recent studies have highlighted the impact of age-related T cell dysregulation on tumor control and immunotherapy outcomes [386,387]. Chen et al. [386] found that in aged mice, tumors grow faster, tumor-infiltrating CD8^+^ T cells are fewer, and the number of ovalbumin-specific CD8^+^ T cells within tumors and tumor-draining lymph nodes is reduced. These data indicate that aging impairs tumor control and extensively alters the fate and function of CD8^+^ T cells. The results of Dahlquist et al. [387]. suggested that PD1 enhances the response of aged mice to normal microbial experience by increasing the cytotoxic capacity of CD8^+^ T cells, thereby improving immunosenescence and extending lifespan. Their results indicate that targeting and reversing immunosenescence may be an effective therapeutic strategy. In addition, in antiaging and anticancer therapies, senolytic drugs and senomorphic drugs are 2 key research directions. The primary function of senolytic drugs is to selectively clear senescent cells. These drugs achieve this by inducing apoptosis in senescent cells [388,389]. The main function of senomorphic drugs is to reduce the harmful effects of senescent cells by altering or modulating their phenotype, rather than necessarily eliminating these cells [390,391]. Table 2 summarizes the drugs targeting senescence in cancer therapy [392–431]. In the future, combining antiaging drugs with chronotherapy to optimize cancer treatment may be a promising direction. On the one hand, conducting pharmacokinetic studies to determine the absorption, distribution, metabolism, and excretion characteristics of senolytic and senomorphic drugs at different times of the day is important. Key parameters such as half-life, peak plasma concentrations, and clearance rates can vary over the course of a day due to circadian fluctuations in physiological processes. Understanding these variations is essential for optimizing dosing schedules and maximizing drug efficacy. In addition, the field of drug delivery through nanocarriers is gaining momentum as it enhances cancer treatment effectiveness while minimizing side effects [432–434]. Combining chronotherapy with nanotechnology shows important promise for safer and more efficient cancer therapies [316,435,436]. Nanocarriers, such as nanoparticles, enable precise drug delivery that aligns with the body’s circadian rhythms, optimizing therapeutic effects by releasing drugs at specific times based on stimuli like pH and temperature [437–439]. On the other hand, biomarker monitoring involves tracking specific molecules or indicators in the body that are associated with aging and drug response. By monitoring these biomarkers, clinicians can determine the optimal times for drug administration. For instance, circadian rhythms of senescence markers such as p16 and p21, as well as inflammatory markers like IL-6 and TNF-α, can be tracked to assess their fluctuations throughout the day. These biomarkers provide valuable insights into the biological processes underlying aging and drug response, allowing clinicians to tailor treatment schedules to maximize efficacy and minimize adverse effects.

**Table 2.** The summary of drugs targeting senescence in cancer therapy

Target senescence drugs	Class	Agents	Mechanism	Preclinical study	Clinical studies	Reference
Senolytic drugs	Bcl-2 family protein inhibitors	ABT-737	↓Bcl-2, ↓Bcl-xL, ↓Bcl-W, ↑apoptosis	Significant antitumor activity in various in vivo and in vitro cancer models, including lymphoma, SCLC, OC, AML, and so on, showing synergy with other chemotherapeutic agents.	NA	[392–395]
		Navitoclax (ABT263)	↓Bcl-2, ↓Bcl-xL, ↓Bcl-W, ↑apoptosis	Significant preclinical efficacy in various cancers, including lymphoma, SCLC, and other solid tumors. Although resistance due to Mcl-1 expression and thrombocytopenia posed challenges, combination strategies and dose management provided effective solutions.	NCT01087151, NCT00878449, NCT01009073, NCT00878449, NCT00891605, NCT00888108, NCT00887757, NCT02591095	[396–402]
		Nav–gal	↓Bcl-2, ↓Bcl-xL, ↓Bcl-W, ↑apoptosis	Selective activation in cells exhibiting high SA-β-gal activities, combined treatments with cisplatin, enhanced antitumor effects in lung cancer xenografts.	NA	[403]
		Dasatinib + quercetin	↓Bcl-xL, ↓PI3K/Akt, ↓p16, ↓p21, ↑apoptosis	Inhibition of tumor progression by depleting senescent hepatic stellate cells. Dasatinib and quercetin combined with carboplatin or olaparib can reduce the peritoneal and adipose tissue metastasis of OC.	NA	[404,405]
	BET family protein inhibitors	ARV-825	↓BET family proteins	Significant antitumor activity in GC, thyroid carcinoma, T-ALL cell line. Sequential administration of doxorubicin followed by ARV825 effectively attenuated tumor growth in a mouse model of obesity-induced HCC.	NA	[406–409]
	Cardiac glycosides	Digoxin	↓Na+/K+ pumps, ↑apoptosis	In various cancer cell lines, including lung, liver, melanoma, breast, and colon, combinations of alisertib, barasertib, tozasertib, palbociclib, or etoposide were investigated. Additionally, bleomycin chemotherapy was explored in lung cancer cell lines, while gemcitabine was tested in lung cancer xenografts and doxorubicin in breast cancer PDX models.	NA	[410,411]
		Ouabain	↓Na+/K+ pumps, ↑apoptosis	In lung, liver, melanoma, breast, and colon cancer cell lines, combinations of alisertib, barasertib, tozasertib, palbociclib, or etoposide were investigated.	NA	[411]
	mTOR inhibitors	AZD8055	↓mTOR pathway	Antiproliferative and apoptotic effects on SCLC, HCC, breast cancer cells, along with antiangiogenic properties, synergistic interactions with other anticancer agents, and efficacy in inhibiting tumor growth and metastasis in animal models.	NA	[412–414]
	Natural products	Fisetin	↓p16, ↓p21, ↑cleaved caspase- 3/7, ↑apoptosis	Fisetin can inhibit cancer cell proliferation, induce apoptosis, and interfere with several key signaling pathways involved in tumor growth and metastasis.	NCT04733534	[415,416]
Senomorphic drugs	NF-κB inhibitors	Metformin	↓NF-κB, ↑DICER1, ↓Akt, ↓p16, ↓p21	Diverse model organisms have proved that metformin is effective in suppressing cellular senescence and SASP.	NCT01101438, NCT01210911, NCT02115464, many trials but unclear if this involves in senescence	[417,418]
		Rapamycin	↓NF-κB, ↓mTOR, ↓p16, ↓p21, ↑autophagy, ↓ROS	Suppression of tumor-promoting effects of senescent cells in mice by reducing SASP.	NCT00707135, NCT00708591, NCT00375245, unclear if this involves in senescence	[419]
	p38MAPK inhibitor	SB203580	↓p38 MAPK	Significant anticancer effects by reducing cell proliferation, inducing apoptosis, inhibiting metastasis, and sensitizing cancer cells to chemotherapy and radiation.	NA	[420–422]
	JAK/STAT inhibitors	Ruxolitinib	↓JAK	Inducing apoptosis and pyroptosis of cancer cells overcomes cisplatin resistance.	NCT02876302	[423–425]
	IL-6 inhibitors	Siltuximab	↓IL-6	Improving the human malignancy therapy, linked to SASP (IL-6) reduction.	NCT00911859, NCT01531998	[426–428]
	IL-1β inhibitors	Canakinumab	↓IL-1β	Improving the human malignancy therapy, linked to SASP (IL-1β) reduction.	NCT03447769, NCT03631199	[429–431]

NA, not available; SCLC, small cell lung cancer; OC, ovarian cancer; AML, acute myeloid leukemia; CLL, chronic lymphocytic leukemia; BET, bromodomain and extraterminal domain; GC, gastric cancer; T-ALL, T-cell acute lymphoblastic leukemia; HCC, hepatocellular carcinoma; PDX, patient-derived xenograft; SASP, senescence-associated secretory phenotype.

Recently, researches on the phenomenon of escape from senescence have gained increasing attention. Escape from senescence refers to the phenomenon where certain cells bypass the state of permanent cell cycle arrest through mechanisms such as genetic mutations, epigenetic alterations, telomerase reactivation, or the influence of the SASP [440–444]. This process has complex implications in biology and pathology; it facilitates cancer initiation and progression, as escaped cells often exhibit genomic instability and unchecked proliferation; for instance, a study demonstrated that chemotherapy-induced senescence in malignant cells can enhance stemness, characterized by the up-regulation of stem cell signatures, activation of Wnt signaling, and acquisition of self-renewing properties. Critically, cells that escape from senescence exhibit increased clonogenic potential and tumor-initiating capacity, both in vitro and in vivo. These findings, corroborated by evidence from human cancer cell lines and primary samples, underscore the plasticity of cancer cells and reveal that senescence-associated stemness may contribute to tumor relapse and progression [441]. Another study [443] revealed that prolonged p21WAF1/Cip1 induction in p53-null models enables a subset of cells to escape senescence, leading to heightened genomic instability, aggressiveness, and chemoresistance. Mechanistically, p21WAF1/Cip1 disrupts replication licensing by saturating ubiquitin ligase complexes, triggering replication stress. Similarly, Zhang et al. [445] found that doxorubicin-induced senescent breast cancer cells promoted epithelial mesenchymal transition, migration, and invasion in adjacent nonsenescent cells through SASP in direct coculture. SASP also facilitated senescent cells to escape senescence, re-enter the cell cycle, and regain tumor characteristics. Mechanistically, Notch signaling was activated in both senescent and nonsenescent cells, driving epithelial–mesenchymal transition (EMT) and senescence escape. Inhibiting Notch signaling with N-[(3,5-difluorophenyl)acetyl]-l-alanyl-2-phenyl]glycine-1,1-dimethylethyl ester (DAPT) blocked EMT and reduced lung metastasis. In summary, understanding the molecular mechanisms behind this phenomenon is crucial for developing targeted cancer therapies and exploring interventions in age-related diseases.

Importantly, exosomes are small extracellular vesicles (sEVs), typically ranging from 30 to 150 nm in diameter, that are secreted by cells [446]. They are formed through the inward budding of the cell membrane and are released into the extracellular environment via a process known as exocytosis [447,448]. Exosomes contain various bioactive molecules, including proteins, lipids, and RNA (such as miRNA and mRNA), which allow them to facilitate intercellular communication and regulate various physiological and pathological processes. They have been implicated in the development and progression of various diseases, including cancer, neurodegenerative disorders, and cardiovascular diseases. Due to their ability to carry tumor markers and other biomarkers, exosomes hold important potential for disease diagnosis, prognosis, and therapy [449,450]. Notably, during the aging process, cells exhibit a significant increase in exosome secretion, particularly in OIS [451,452]. This may be related to the role of senescent cells in influencing the surrounding microenvironment through exosomes, transmitting aging signals, or enhancing exosomes secretion as a mechanism to maintain cellular homeostasis. Furthermore, senescent cells can play an important role in the tumor microenvironment by secreting exosomes containing the SASP. A recent study [451] showed that exosome-like sEVs are critical mediators of the pro-tumorigenic functions of senescent cells. Specifically, EphA2 protein, which is sorted into sEVs secreted by senescent cells, can bind to ephrin-A1, a ligand highly expressed in various cancer cells, thereby promoting cancer cell proliferation through EphA2/ephrin-A1 reverse signaling. Further research reveals that the enhanced phosphorylation of EphA2, due to oxidative inactivation of the PTP1B phosphatase in senescent cells, leads to its increased sorting into sEVs. This finding uncovers a novel mechanism where reactive oxygen species regulate cargo sorting into sEVs, which plays a crucial role in the potentially deleterious growth-promoting effects of the senescent cell secretome. This mechanism offers new insights into how the secretome of senescent cells influences cancer progression. Future research could focus on developing therapies that target exosomes released by senescent cells to block the spread of SASP factors. This approach may help inhibit tumor progression and reduce the risk of malignancies associated with aging. Drugs that can specifically inhibit exosome production, release, or uptake, or modify the exosomal cargo through genetic engineering, could become a crucial component of next-generation cancer therapies. In addition, since exosomes can be detected in bodily fluids (such as blood and urine) and their cargo reflects changes in the tumor microenvironment, characterizing SASP factors within exosomes could lead to new biomarkers for early cancer detection. This noninvasive diagnostic method could be more convenient and safer than traditional tissue biopsies.

However, current research also has limitations. Due to the diverse microenvironmental stimuli and physiological functions of different cell types, senescent cells exhibit spatiotemporal heterogeneity. This may be due to the diversity in factors such as age, gender, pathological states, tissue location, microenvironment, and accumulation dynamics. Therefore, it is especially important to gain a deeper understanding of the heterogeneity of senescent cells and to develop targeted therapeutic models and interventions. Although factors like p16 and p21, which regulate the cell cycle, are widely regarded as conserved markers of senescent cells, recent studies have found that these markers are not applicable to all human cells or tissues [234,453,454]. For example, research has found that some cells with high p16 expression, such as pancreatic β-cells, macrophages, and mesenchymal stem cells, do not exhibit senescent characteristics [455–457]. Moreover, not all senescent cells show high levels of p16 [234]. Currently, with technological advancements, studying the heterogeneity of senescence through single-cell sequencing has become very common [234,458,459]. Additionally, single-nucleus RNA sequencing, spatial transcriptomics, multiplexed antibody imaging, multiplex fluorescence in situ hybridization, flow cytometry imaging, and single-cell mass spectrometry are also being gradually applied to the study of senescence heterogeneity, demonstrating marked potential. In summary, a key issue that urgently needs addressing is the development of reliable, sensitive, and specific identification methods to accurately determine the location of senescent cells in tissues and quantify their abundance both in vitro and in vivo. In this respect, organic nanoprobes have demonstrated important potential in detecting senescent cells, leveraging their exceptional optical and chemical properties to accurately identify and label these cells [460,461]. This capability is critical for studying the mechanisms of cellular senescence and related diseases. A recent study [462] describes a biocompatible, injectable organic nanoprobe called NanoJagg. NanoJaggs are high-purity indocyanine green dimer nanostructures that can specifically detect senescent cells through fluorescence and photoacoustic imaging. Their simple synthesis and strong photoacoustic tomography signal make them promising for clinical applications. Monitoring in vivo senescence burden using NanoJaggs can provide crucial insights into tissue dysfunction and improve disease diagnosis and risk stratification, including for cancer. In addition, Magkouta et al. [463] developed and validated a novel fluorescent dye, GLF16, and its micelle vector for precise labeling and analysis of senescent cells in vivo and in vitro. GLF16, a fluorescent Sudan Black-B analog, efficiently detects senescent cells using fluorescence microscopy and flow cytometry, while the micelle vector enhances the uptake of GLF16 by senescent cells in living organisms and cultures. This method significantly improves the isolation and real-time tracking of senescent cells, providing an innovative tool for in-depth exploration of aging-related mechanisms. Moreover, natural aging animal models are expensive and have long experimental cycles, while transgenic models do not fully mimic true aging processes. Therefore, studying cellular senescence in animal models has limitations, highlighting the need for new models and senescent cell culture systems that better represent human aging for deeper exploration.

### Development and validation of circadian-related biomarkers: New opportunities

In addition, it is important to identify novel circadian-related biomarkers for early detection, prognosis, and monitoring of aging-related diseases and cancer. By leveraging advanced technologies such as genomics, proteomics, and metabolomics, researchers could discover novel biomarkers associated with circadian rhythms, aging, and tumorigenesis [464]. These biomarkers hold the potential to revolutionize clinical practice by enabling more accurate disease diagnosis, risk stratification, and treatment response prediction. For instance, the expression profiles of circadian clock-related genes have been shown to have prognostic implications in non-small cell lung cancer, where a 10-gene signature could independently predict overall survival [465]. Similarly, circadian clock gene signatures have been used to identify high-risk early-stage lung adenocarcinoma patients, demonstrating their potential as prognostic biomarkers [466]. Moreover, the circadian rhythm pathway has been associated with prostate cancer progression, where genetic variants in circadian genes such as NPAS2 have been linked to disease progression [467]. This suggests that circadian genes could serve as biomarkers for monitoring cancer progression and potentially guiding treatment decisions.

Importantly, lifestyle interventions involve the adoption of behaviors and habits that align with circadian rhythms to promote health aging and prevent cancer. These interventions encompass practices such as maintaining regular sleep–wake cycles, incorporating physical activity into daily routines, and following balanced nutrition plans. By emphasizing the importance of circadian-friendly lifestyle choices, educational programs and public health initiatives seek to raise awareness and empower individuals to make informed decisions about their health. Through the implementation of lifestyle interventions, we aim to optimize circadian alignment, mitigate the adverse effects of circadian disruption, and promote overall well-being, ultimately reducing the risk of aging-related diseases and cancer and improving long-term health outcomes.

## Conclusion

In this review, we initially outlined the physiological underpinnings of circadian rhythms and gave a general review of how these rhythms affect the genesis and spread of tumors. Next, we discussed the possible processes and relationships between aging and circadian rhythms. We then concentrated on the hallmarks that are shared by cancer and aging, such as cellular senescence, chronic inflammation, epigenetic changes, and genomic instability. Then, we conducted a thorough analysis of the common hallmarks and interactions between cancer, aging, and circadian rhythms. These interactions included carcinogenesis, apoptosis and tumor growth, genomic instability, cellular senescence, and cellular metabolism. Finally, we offered insights into translating current research findings into clinical practice, focusing on chronotherapy and antiaging treatments by integrating immunotherapy, senescent cell detection, and cutting-edge nanocarrier delivery systems.

## Ethical Approval

The authors are accountable for all aspects of the work in ensuring that questions related to the accuracy or integrity of any part of the work are appropriately investigated and resolved.
